# Integration of network pharmacology, transcriptomics and single-cell sequencing to explore the effect of Rougan Keli in alleviating liver cirrhosis

**DOI:** 10.1186/s13020-025-01220-z

**Published:** 2025-10-02

**Authors:** Chengbo Jin, Tianle Ma, Yiheng Zhang, Xujing Gu, Tong Zhu, Xinyu Wu, Xin Ding, Suzhou Huang, Yulan Wang, Zhipeng Chen, Huihua Fang, Li Wu

**Affiliations:** 1 Jiangsu Key Laboratory for Pharmacology and Safety Evaluation of Chinese Materia Medica, School of Pharmacy, Nanjing University of Chinese Medicine, Nanjing, 210023 China; 2 College of Pharmaceutical Sciences, Soochow University, Suzhou, 215123 Jiangsu China; 3 Jiangsu Province Hospital of Chinese Medicine, Affiliated Hospital of Nanjing University of Chinese Medicine, Nanjing, 210004 China

**Keywords:** Rougan Keli, Transcriptomics, Single-cell sequencing, Liver cirrhosis, HSC, LSEC

## Abstract

**Background:**

The progression of liver cirrhosis leads to severe complications, significantly threatening the survival and prognosis of patients. Rou gan keli (Rgkl), a herbal formula derived from classical prescriptions, has used clinically over two decades and has good efficacy. However, its molecular mechanisms and active components remain undefined.

**Purpose:**

Exploring the molecular mechanisms of Rgkl in alleviating liver cirrhosis.

**Methods:**

CCl_4_-induced liver cirrhosis mice models were established. Liver stiffness and intrahepatic blood flow velocity were assessed using imaging. Serum ALT, AST, HA, and histopathology were analyzed. Hepatic stellate cells (HSCs) activation, liver sinusoidal endothelial cells (LSECs) fenestration, and angiogenesis were evaluated using immunohistochemistry and scanning electron microscopy. UPLC-Q-TOF-MS/MS and network pharmacology identified active components. Transcriptomics and single-cell sequencing identified key targets and pathways, validated via WB, immunofluorescence, and molecular docking.

**Results:**

Rgkl significantly reduced Liver stiffness and collagen deposition while increasing intrahepatic blood flow velocity in cirrhotic mice. Serum ALT, AST, and HA were markedly decreased. Rgkl inhibited α-SMA expression in HSC and downregulated pathological angiogenesis by reducing VEGF and CD34 expression. Additionally, Rgkl enhanced eNOS expression and preserved sinusoidal fenestration in LSEC. Furthermore, Rgkl ameliorated liver cirrhosis by modulating LSEC metabolic functions via the CD36/PPAR/CPT-1 pathway and suppressing HSC activation through the RhoA/ROCK/YAP and PI3K/AKT/NF-κB pathways. Eighteen active components, such as Levistilide A and Quercetin, were strongly correlated with the amelioration of liver cirrhosis.

**Conclusions:**

Rgkl significantly attenuated hepatic injury and fibrosis. Mechanistically, Rgkl modulated LSEC lipid metabolism and phenotypic regulation, and suppressed HSC contraction and activation. Key active components contributing to these effects included Paeonilactone C, Levistilide A, and Quercetin.

**Graphical Abstract:**

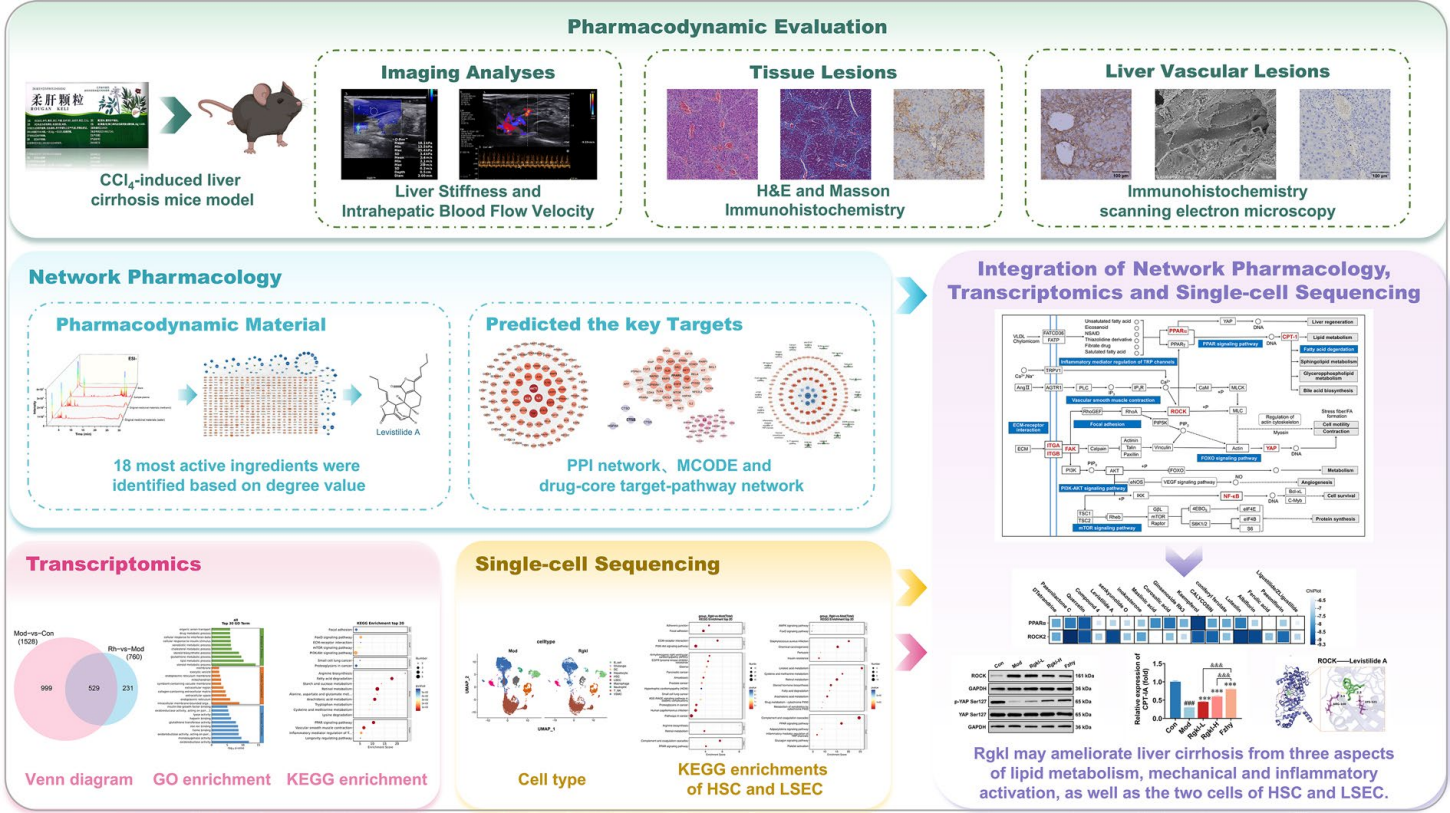

**Supplementary Information:**

The online version contains supplementary material available at 10.1186/s13020-025-01220-z.

## Introduction

Liver cirrhosis represents the terminal pathological stage of various chronic liver diseases, with delayed or ineffective treatment often leading to life-threatening complications such as portal hypertension (PH), ascites, upper gastrointestinal bleeding, and hepatocellular carcinoma. Epidemiological studies reveal that approximately 1.16 million global deaths annually are attributable to liver cirrhosis, with Chinese patients accounting for 11% of this mortality burden [1], highlighting the urgent need for effective therapeutic strategies to mitigate disease progression and improve patient prognosis. PH, the most prominent complication in end-stage liver cirrhosis, is primarily driven by persistently elevated intrahepatic vascular resistance (IHVR). This pathophysiological alteration stems from extensive hepatocyte necrosis, diffuse nodular regeneration, and fibrotic septa formation, which collectively promote pseudolobule formation. These structural derangements result in hepatic architectural collapse and marked distortion of hepatic vascular architecture [2], severely impeding portal venous return. Moreover, in addition to extensive parenchymal necrosis, non-parenchymal cells—specifically liver sinusoidal endothelial cells (LSECs) and hepatic stellate cells (HSCs)—play decisive roles in elevating intrahepatic vascular resistance (IHVR). Under pathological conditions, LSEC defenestration and basement membrane formation critically impair hepatic blood supply and metabolic exchange. Furthermore, LSECs secrete vascular endothelial growth factor (VEGF) to promote pathological angiogenesis, exacerbating hepatic vascular network disorganization. Notably, LSEC-derived endothelin-1 (ET-1) induces aberrant HSC contraction, which mechanically distorts vascular walls and increases intrahepatic hemodynamic resistance. Concurrently, activated HSCs exacerbate IHVR through excessive extracellular matrix (ECM) deposition, which elevates tissue stiffness and compresses sinusoids. Recent studies have highlighted the critical role of HSC contraction and mechanotransduction pathways in liver cirrhosis progression. Emerging evidence demonstrates that both mechanical and biochemical cues activate the RhoA/ROCK signaling cascade in HSCs, driving stress fiber assembly and sustained cellular contractility. Concurrently, mechanosensitive YAP/TAZ signaling in fibrotic liver tissues promotes COL1A1 secretion, establishing a self-reinforcing “COL1A1-tissue stiffness-YAP” tumor-promoting microenvironment [3, 4]. Additionally, elevated IHVR directly disrupts LSEC sphingolipid metabolism, upregulating the carcinogenic mediator sphingosine-1-phosphate (S1P). These findings collectively underscore that LSECs and HSCs synergistically drive liver cirrhosis through multifaceted mechanisms, emphasizing the necessity for multi-target therapeutic strategies rather than single-cell or mono-pathway interventions.

Rgkl was developed based on the therapeutic principle of the classical formula Xuefu Zhuyu Decoction [5], particularly inspired by its representative herb pair *Angelica sinensis* (Oliv.) Diels (Danggui, DG) and *Paeonia lactiflora* Pall. (Chishao, CS), which are renowned for their blood-activating and stasis-resolving properties. To enhance its efficacy, Rgkl incorporates additional medicinal herbs, including *Panax notoginseng* (Burkill) F.H.Chen (Sanqi, SQ), *Lycopus lucidus* Turcz. ex Benth. (Zelan, ZL), *Ligustrum lucidum* W.T.Aiton (Nüzhenzi, NZZ), *Achyranthes bidentata* Blume (Niuxi, NX), *Astragalus membranaceus* (Fisch.) Bunge (Huangqi, HQ), *Atractylodes macrocephala* Koidz. (Baizhu, BZ), and *Stephania tetrandra* S.Moore (Fangji, FJ). As a hospital-approved formulation (Approval No. Su-Yao-Zhi-Z04000542) of Jiangsu Provincial Hospital of Traditional Chinese Medicine (TCM), Rgkl has been clinically utilized for over two decades to treat liver cirrhosis and its complications. Among them, DG and CS act as principal drugs, softening the liver and nourishing blood. ZL and SQ enhance blood circulation and dispel stasis, while NX and NZZ support liver and kidney functions as minister drugs. HQ, BZ, and FJ, serving as adjuvant drugs, strengthen the spleen (soil in TCM) and the liver (wood in TCM), and promote diuresis. This combination aims to soften the liver, bolster the spleen, stimulate blood flow, and clear collaterals, primarily targeting liver cirrhosis characterized by vital energy deficiency and blood stasis in the liver and spleen. Clinical studies and experiments have demonstrated Rgkl's efficacy in significantly improving portal vein flow velocity in liver cirrhosis patients, potentially normalizing it, and alleviating PH. Notably, DG has been identified as effective in reducing PH [6], while CS can ameliorate liver tissue pathology, decrease IHVR, protect hepatocytes, and exhibit anti-fibrotic properties [7]. HQ and FJ have also been shown to effectively dilate blood vessels and reduce IHVR [8]. However, the primary active components and molecular mechanisms by which these drugs modulate liver sinusoidal pressure and relieve PH in liver cirrhosis remain to be elucidated.

## Materials and methods

### Reagents

The chemicals are listed as follows: Rgkl (Formula is listed in Table. S1) was purchased from Jiangsu Provincial Hospital of traditional Chinese medicine; Fuzheng huayu capsule (Fzhy) (No. of National Medicine: Z20020074) was purchased from Shanghai Huanghai Pharmaceutical Co., Ltd. HPLC grade acetonitrile and methanol (produced by Merck, Germany). Formic acid (mass spectrometry grade, Merck, Germany); Ultrapure water (laboratory Millipore ultrapure water); Olive oil (O108686; aladdin, Shanghai, China); CCl_4_ Stock solution (C119833; aladdin, Shanghai, China); D-Tetrandrine (HY-13764; MedChemExpress, New Jersey, USA); Levistilide A (RS06581020; Shanghai Standard Technology Co.,Ltd); Corosolic acid (HY-N0280; MedChemExpress, New Jersey, USA); Ginsenoside Rk3 (HY-N0906; MedChemExpress, New Jersey, USA); Paeoniflorin (HY-N0293; MedChemExpress, New Jersey, USA); CCK-8 (ZYCD002-0100; ZUNYAN, Nanjing, China).

The primary antibodies are listed as follows: α-SMA (MB3218; Bioworld, Nanjing, China); CD34 (CY5196; Abways, China); CPT-1A (K000391P; Solarbio^®^, Beijing, China); eNOS (27120-1-AP; proteintech^®^, Wuhan, China); GAPDH (AF7021; Affinity, Jiangsu, China); Integrin alpha 4 (DF6135; Affinity, Jiangsu, China); Integrin beta 1 (AF5379; Affinity, Jiangsu, China); NF-κB p65 (10745-1-AP; proteintech^®^, Wuhan, China); PI3K p85 alpha (60225-1-Ig; proteintech^®^, Wuhan, China); ROCK2 (K006732P; Solarbio^®^, Beijing, China); VEGF (DA019; Novoprotein,, Shanghai, China); YAP (30323; Promab); YAP-Ser127 (AP0489; ABclonal Technology, Wuhan, China).

### Cell lines

The Hepatic stellate human cell LX-2 were obtained from Shanghai ZhongQiao Xin ZhouBiotechnologyCo.,Ltd. (ZQ0026) and were cultured at 37 ℃ with 5% CO_2_ in RPMI 1640 Medium (KGL1501-500, KeyGEN BioTECH) supplemented with 10% Fetal Bovine Serum (FBS) (209111, NEST Biotechnology) and Penicillin–Streptomycin Solution (BC-CE-007, BioChannel Biological Technology Co., Ltd.). These cell lines were authenticated by examination of morphology and consistent in vivo performance. Culture bottles and dishes (CCB06-025, CCB06-075, and CCD06-100A) were purchased from Bioland Biotechnology.

### Preparation of Carbon tetrachloride solution and drug solution

#### CCl_4_ solution

A 98% pure CCl_4_ stock solution was aspirated and dissolved in pure olive oil to formulate a 20% CCl_4_ modeling solution (3 mL CCl_4_ + 12 mL olive oil), which was mixed and then used for intraperitoneal injection in mice at a dose of 5 mL/kg [9]. In addition, the control group was given an equal amount of olive oil for simultaneous intraperitoneal injection (5 mL/kg). The molding cycle was 10 weeks, and injections were given twice a week.

#### Rgkl suspension

The human clinical dose of Rgkl is 1 g/kg, so the clinical equivalent dose for mice is approximately 9 g/kg. Take the clinical equivalent dose of mice as Rgkl-L, and double the clinical equivalent dose as Rgkl-H (18 g/kg). According to the gavage volume of 0.2 mL/10 g, the concentrations of Rgkl-L and Rgkl-H were calculated to be 0.45 g/mL and 0.9 g/mL, respectively. The specific configuration method was to dissolve 9 g of Rgkl in 10 mL of ultrapure water and use ultrasound assisted dissolution to obtain a high-dose Rgkl of 0.9 g/mL. Subsequently, mixed 3 mL of high-dose Rgkl with 3 mL of ultrapure water and diluted to obtain low-dose Rgkl.

#### Fzhy suspension [10, 11]

The human clinical dose of Fzhy is 0.1 g/kg, so the clinical equivalent dose for mice is approximately 0.9 g/kg. According to the gavage volume of 0.2 mL/10 g, the concentration of Fzhy should be 0.045 g/mL. The specific configuration method was to dissolve one capsule content (0.5 g) in 11.1 mL of ultrapure water and use ultrasound assisted dissolution.

#### UPLC‒MS/MS analysis of Rgkl blood components

Sprague–Dawley rats (180–200 g) were acquired from Nanjing Anokang Biotechnology Co., Ltd. (certificate no. SCXK (LU) 20220006). The rats were housed in a facility regulated for temperature and humidity, under a 12 h light/dark cycle, and in pathogen-free conditions, with free access to rodent chow and water. All experimental protocols were approved by the Animal Ethics Committee of Nanjing University of Traditional Chinese Medicine (ethical approval no. 202306A062). Rats were acclimatised for one week and then randomly assigned to either a control group or a treatment group (Rgkl, 12 g/kg), with six rats in each group. The treatment was administered daily for 7 days. Prior to the final dose, the rats underwent a 12 h fast, though water remained accessible. 1 h after the last dose, the rats were anesthetized with an intraperitoneal injection of 10% pentobarbital sodium at a dose of 3.5 mL/kg. Blood was then collected from the abdominal aorta, allowed to stand at room temperature for 1 h and subsequently centrifuged for 10 min at 4 ℃ and 3500 rpm to obtain the supernatant.

A 100 μL plasma sample was combined with 300 μL methanol, vortexed for 3 min, and centrifuged for 5 min at 12000 rpm. The supernatant was then dried under nitrogen and reconstituted in 100 μL methanol, followed by vortexing for 3 min. After a further centrifugation for 5 min at 12000 rpm, 1 μL of the supernatant was analyzed using UPLC-Q-TOF-MS/MS. Chromatographic separation was achieved on an Agilent C_18_ reversed-phase column (2.1 mm × 100 mm, 1.8 μm) with an Agilent C_18_ guard column. The mobile phases consisted of 0.1% formic acid (A) and acetonitrile (B). The elution gradient was as follows: 0–4 min, 5–25% B; 4–14 min, 25–65% B; 14–20 min, 65–80% B; 20–24 min, 80–95% B; 24–28 min, 95–5% B; and 28–30 min, 5% B, at a flow rate of 0.3 mL/min. The column temperature was maintained at 35 °C, and the injection volume was 1 μL. For mass spectrometry, an ESI source was used in both positive and negative ion modes with the following settings: ion spray voltage floating (ISVF) at 4500/− 4500 V, temperature (TEM) at 550 °C, declustering potential (DP) at 60/− 60 V, collision energy (CE) at 35/− 35 eV, nitrogen as the nebulizing gas, with Gas 1 and Gas 2 both set to 55 psi, and the curtain gas at 35 psi. The Q1 and Q2 scan ranges were m/z 100–2000 and m/z 50–1000, respectively, with dynamic background subtraction (DBS) enabled.

To identify the compounds present in Rgkl, literature searches were conducted using CNKI and PubMed databases. Molecular files for each compound were downloaded to calculate accurate mass-to-charge ratio values for ion species. Raw mass spectrometry data were processed in Peak View software, with chemical structures confirmed by matching secondary fragment ions from each compound's total ion chromatogram against those in the corresponding molecular files. Compounds were considered successfully identified with a mass error < 10 ppm and a match score of ≥ 60%.

#### Animal experiments

SPF grade C57BL/6 J male mice 6 weeks old, provided by Beijing Viton Lihua Laboratory Animal Technology and Wood Co., Ltd. certificate no. [SCXK (Beijing) 20210006]. They were kept in the Animal Experiment Center of Nanjing University of Traditional Chinese Medicine, and the experimental animal procedures were in accordance with the ethical regulations (Ethical Approval No. H0011230105572123). 50 mice were taken and randomly divided into control group, model group, Rgkl low dose group (Rgkl-L), Rgkl high dose group (Rgkl-H) and Fzhy group. The control group was injected intraperitoneally with olive oil, and the model group was injected intraperitoneally with 20% CCl_4_. After 2 weeks of modeling, the Rgkl-L, Rgkl-H, and Fzhy groups were started to be administered by gavage. The pathological changes of hepatic fibrosis and liver cirrhosis were confirmed using HE staining at the 4th and 8th week of the experiment, respectively. At the end of the experiment, blood was taken and serum was separated to check the transaminase level and fibrosis serological indexes, and the liver was taken, weighed, and an appropriate amount of liver tissue was placed in 4% formaldehyde, 2.5% glutaraldehyde fixative, or stored in a refrigerator at − 80 °C, respectively, to detect the tissue lesions.

#### Primary LSEC and HSC isolation and culture

Male C57BL/6 J mice (6–8 weeks) were fasted, anesthetized with sodium pentobarbital (50 mg/kg), and disinfected. The portal vein was cannulated with a 24G needle for perfusion. Pre-perfusion with heparinized Phosphate-Buffered Saline (PBS, 0.5 mM EDTA, 37 °C) removed blood, followed by enzymatic digestion with Hank’s Balanced Salt Solution (HBSS, 37 °C) containing 0.05% Collagenase Type IV (collagenase IV) and 0.005% Deoxyribonuclease I (DNase I) until liver softening. The excised liver was minced in ice-cold HBSS, digested in HBSS/DNase I with agitation, and filtered through sequential strainers (100 μm/70 μm).

Hepatocytes were removed by low-speed centrifugation; non-parenchymal cells (NPCs) were retained. NPCs were layered onto discontinuous Nycodenz gradients (25%/50% and 8.2%/17% in HBSS) and centrifuged. LSECs from the 25–50% interface and HSCs from the 8.2–17% interface were collected and washed. LSECs were cultured in Dulbecco’s Modified Eagle Medium (DMEM) with 10% FBS, 1% penicillin/streptomycin, and 1% endothelial cell growth supplement (ECGS), adhering with cobblestone morphology. HSCs in DMEM/10% FBS exhibited lipid droplets and vitamin A autofluorescence under UV light.

#### Automatic biochemistry analyser to detect biochemical indicators

Blood was taken from mouse orbits, about 0.5 mL from each, left at room temperature for 1 h, centrifuged at 4 ℃, 3500 rpm for 10 min, and the supernatant was obtained. ALT and AST were detected by Roche Biochemistry and Immunology Pipeline Biochemistry Module (cobas, c701), and HA, PC-III and PC-IV were detected by fully automated chemiluminescence immunoassay analyser (MAGLUMI X3).

#### Type B ultrasonic inspection

Liver stiffness values and hepatic blood flow velocity were examined at weeks 6 and 10 of the experiment, respectively. The mice were deprived of abdominal hair and fasted from food and water the night before the test (6:00 PM). They were placed on the examination bed and the limbs were compressed during the test. Measurements were performed with Ultrasonic Imaging Diagnostic Device (Aixplorer V) with frequency settings of 7.5–10 MHz.

#### Histological hematoxylin–eosin and masson staining

Liver specimens were pre-served in 4% paraformaldehyde and then dehydrated in a graded alcohol series. Then, the sections were embedded in paraffin blocks, cut into 5 μm-thick sections and placed on glass slides and were stained with hematoxylin–eosin and masson for histopathological study at last. HE and masson images from 3 fields of each section were taken and quantified with Image J software randomly.

#### Immunohistochemistry staining

Briefly, the tissue sections were deparaffinized with xylene, and rehydrated through graded series of ethanol solutions until water. After antigen retrieval with sodium citrate antigen retrieval buffer and endogenous peroxidase activity quenching, the sections were blocked with 5% bovine serum albumin solution for 30 min and then incubated with indicated primary antibodies (α-SMA, CD34, eNOS and VEGF) overnight at 4℃. Subsequently, the sections were incubated with the corresponding secondary antibodies for 1 h at room temperature. The sections were counterstained with hematoxylin before being dehydrated with the increasing concentrations of ethanol and mounted with neutral resin. The stained sections were imaged using Mantra Pathology Workstation, and the images were analyzed using Image J software.

#### Scanning electron microscope to observe the size and number of hepatic sinusoids

About 1 mm^2^ of fresh tissue blocks were taken within 1–3 min, stored in 2.5% glutaraldehyde fixative solution for 2 h, then placed in 4% osmium buffer solution for 1 h, and then dehydrated by a series of ethanol and permeabilized by butanol. Then the tissue was freeze-dried and smeared with ion sputtering reagent. Finally, the fenestration structure of liver sinusoidal endothelium was quantitatively analyzed by scanning electron microscope, and the data was processed by Image J software to measure its pore size (μm).

#### Western blot analysis

The total protein was extracted by RIPA reagent and determined by BCA assay kits (WB6501; New Cell & Molecular Biotech). Appropriate amounts of SDS loading buffer were added and boiled. The proteins were separated by electrophoresis and electro-transferred to PVDF membranes (SW120; Seven/AbcellsBeijing, China). Then membranes were washed in TBST solution and blocked for 1 h, followed by incubating with primary antibodies. Then the secondary antibody was added and incubated for 2 h. The bands were scanned by Odyssey Western blot machine and the levels of relative proteins were quantitatively analyzed.

#### Network pharmacology

The TCMSP database (https://www.tcmsp-e.com/) and the SwissTarget Prediction database (http://swisstargetprediction.ch/) were utilized to identify the targets of the blood components in Rgkl. The results were consolidated and redundancies minimized. Cirrhosis-related targets were sought in the GeneCards (https://www.genecards.org/) database, OMIM (https://www.omim.org/) database, and DisGeNET (https://www.disgenet.org/) databases.

The overlapping targets were considered potential Rgkl targets for liver cirrhosis treatment. Protein‒protein interaction (PPI) data were obtained from the STRING (https://cn.string-db.org/) database, filtering for nodes with a correlation value greater than 0.4. This data was imported into Cytoscape 3.7.1 software to generate the PPI network, and Network Analyzer was employed to analyze the network's topological features. Additionally, Cytoscape 3.7.1 was used to construct the compound-target network. GO and KEGG pathway enrichment analyses for core targets were performed using the DAVID (https://david.ncifcrf.gov/) database. The selection criteria included a false discovery rate (FDR) of less than 0.01. The results were visualized using the Bioinformatics (https://www.bioinformatics.com.cn/) website and ChiPlot (https://www.chiplot.online/). The above databases were confirmed in authoritative reference [12].

#### Transcriptomics analysis

Liver tissue samples were placed in dry ice and sent to OE Biotech Co., Ltd. (Shanghai, China) for transcriptome sequencing and analysis after the experiment. Total RNA was extracted using the TRIzol reagent (Invitrogen, CA, USA) according to the manufacturer’s protocol. RNA purity and quantification were evaluated using the NanoDrop 2000 spectrophotometer (Thermo Scientific, USA). RNA integrity was assessed using the Agilent 2100 Bioanalyzer (Agilent Technologies, Santa Clara, CA, USA). Then the libraries were constructed using VAHTS Universal V6 RNA-seq Library Prep Kit according to the manufacturer’s instructions. The libraries were sequenced on an llumina Novaseq 6000 platform and 150 bp paired-end reads were generated. Raw reads of fastq format were firstly processed using fast and the low quality reads were removed to obtain the clean reads. The clean reads were mapped to the reference genome using HISAT2. FPKM of each gene was calculated and the read counts of each gene were obtained by HTSeq-count. PCA analysis were performed using R (v 3.2.0) to evaluate the biological duplication of samples. Differential expression analysis was performed using the DESeq2. Q value < 0.05 and foldchange > 2 was set as the threshold for significantly differential expression gene (DEGs). Hierarchical cluster analysis of DEGs was performed using R (v 3.2.0) to demonstrate the expression pattern of genes in different groups and samples. Based on the hypergeometric distribution, GO and KEGG pathway enrichment analysis of DEGs were performed to screen the significant enriched term using R (v 3.2.0), respectively.

These sequencing data were submitted to NCBI GEO database with number GSE272222.

#### Single‐cell RNA‐seq data preprocessing

The sequencing of liver tissue in mice with cirrhosis was completed by OE Biotech Co., Ltd. (Shanghai, China). The scRNA‐seq files were stored in NCBI GEO database with number GSE305510. FASTQ files were aligned to the human genome (MobiVision v3.2), with UMI counts per barcode. The UMI matrix was analyzed in Seurat (v4.0.0). Low-quality cells were filtered using thresholds: genes < 200, UMIs < 1000, log10(Genes/UMI) < 0.7, mitochondrial UMIs > 10%, and hemoglobin UMIs > 5%. Potential doublets were removed using DoubletFinder (v2.0.3). Data were normalized via LogNormalize (scale factor = 10,000) and log-transformed. Top 2000 highly variable genes (HVGs) were selected (Seurat’s FindVariableGenes). PCA was performed, followed by graph-based clustering (FindClusters) and visualization via UMAP (RunUMAP).

Cluster marker genes and differentially expressed genes (DEGs) were identified using FindAllMarkers and FindMarkers (presto test; thresholds: p < 0.05, log2FC > 0.58). GO and KEGG pathway enrichment analyses were conducted via hypergeometric tests in R (v4.0.3).

#### Plasmid construction and cell transfection

The human α-SMA overexpression plasmid (designated as Acta2-OE: 5′-3′ GGCTTATCGAAATTAATACGACTCA) and knockdown plasmid (designated as siActa2: 5′-3′ GCGUGAGAUUGUCCGGGACAUTT), as well as the ROCK overexpression plasmid (designated as ROCK-OE: 5′-3′ AGGTATCTGTACATGGTA

AT) and knockdown plasmid (designated as siROCK: 5′-3′ GUUAGAAACCUGA

CAUUAATT), were constructed by Nanjing KeyGen Biotech Co., Ltd. (Nanjing, China).

Cells were seeded into culture plates and allowed to reach 80% confluence prior to transfection. For transfection, a mixture containing 500 μL of serum-free medium, 4.0 μg of plasmid DNA, and 8 μL of transfection reagent was prepared and incubated at room temperature for 30 min. Subsequently, 1500 μL of serum-free medium was added to the mixture, which was then gently mixed and applied to the cells. After 8 h transfection, the medium was replaced with complete growth medium. mRNA expression levels of the target genes were analyzed 24 h post-transfection to confirm transfection efficiency.

#### Molecular docking

The PDB file of protein target was obtained from PDB database. After H_2_O was removed by Pymol, H was added to AutoDock Vina and converted into pdbqt file. Download SDF files of inhibitor and monomer components of traditional Chinese medicine from PubChem, convert them into PDB format with OpenBabel, and then convert them into pdbqt format files through AutoDock Vina. Molecular docking was simulated by using AutoDock Vina, and the docking targets of inhibitors/agonists and receptor proteins were selected as control. Finally, Pymol is used for visual analysis.

#### Statistical analysis

The results presented in this study were expressed as the mean ± standard deviation (mean ± SD). The statistical method used for comparing the two groups involved the use of Student's t-test, while comparisons among several groups were assessed using analysis of variance (ANOVA) with GraphPad Prism (Version 8.0, San Diego, USA). P values were denoted in figures as: not significant [ns], ^#^*P* < 0.05, ^##^*P* < 0.01, ^###^*P* < 0.001, ^*^P < 0.05, ^**^P < 0.01, ^***^P < 0.001.

## Results

### Effect of Rgkl on imaging and histopathology in cirrhotic mice

**Fig. 1:**
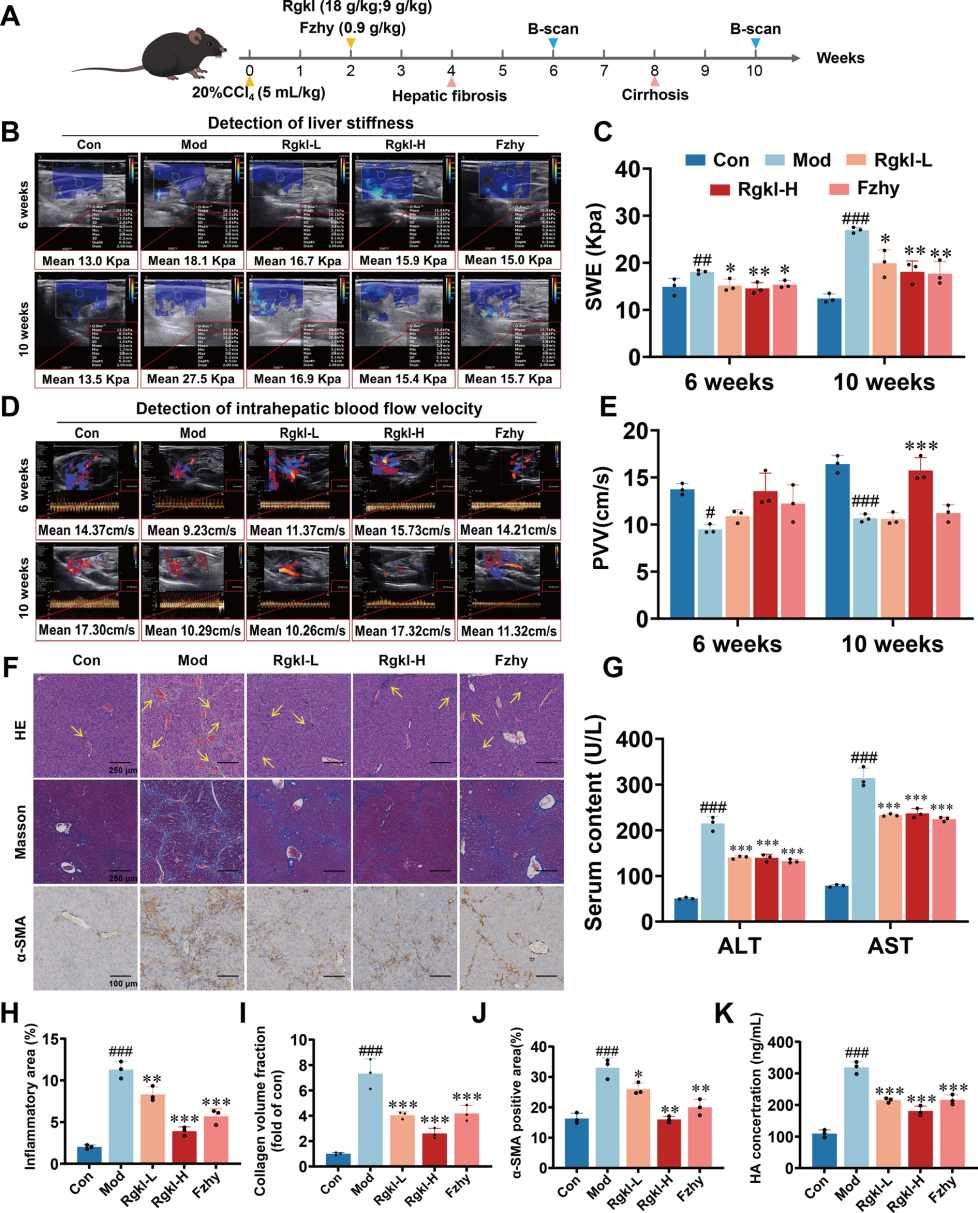
Effect of Rgkl on imaging and histopathology in cirrhotic mice. **A** Animal protocol diagram. **B**, **C** Liver hardness measured at the 6th and 10th weeks. **D**, **E** Intrahepatic blood flow velocity assessed at the 6th and 10th weeks. **F** HE and Masson staining of liver tissue (scale bar = 250 μm) and detection of α-SMA expression by immunohistochemistry (scale bar = 100 μm). **G** Detection of ALT and AST levels in serum. **H** Detection of inflammatory infiltration area. **I** Detection of Masson staining collagen positive area. **J** Detection of α-SMA positive area. **K** Detection of HA content in serum. Data are presented as mean ± SD (3 mice per group). ^*#*^*P* < *0.05, *^*##*^*P* < *0.01, *^*###*^*P* < *0.001* compared with the control group; ^*^P < 0.05, ^**^P < 0.01, ^***^P < *0.001* compared with the model group

In order to evaluate the effect of Rgkl on the progression of liver cirrhosis in mice, imaging analyses were performed at the 6th and 10th weeks of the experiment to dynamically observe the changes in liver hardness values and intrahepatic blood flow velocity (Fig. 1A). It was found that at the stage of liver fibrosis at the 6th week, the model group exhibited a notable increase in liver hardness (Fig. 1B, C; P < 0.01) and a marked reduction in intrahepatic blood flow velocity (Fig. 1D, E; *P* < *0.05*). By the 10th week, the liver stiffness value reached nearly twice that of the control group, and the hepatic blood flow velocities were reduced by more than 30% (*P* < *0.001*). Treatments with low and high doses of Rgkl were able to significantly reduce the liver stiffness value at the stage of hepatic fibrosis (Fig. 1. B, C; *P* < *0.05, P* < *0.01*) and show substantial benefits at the stage of liver cirrhosis (*P* < *0.05, P* < *0.01*), which was also similar to the effect of Fzhy. Furthermore, the high dose of Rgkl exhibited a good improvement effect on intrahepatic blood flow velocity in cirrhotic mice (Fig. 1D, E; *P* < *0.001*) and even close to the level of control group, which was an advantage that Fzhy did not have.

Liver specimens were collected for histopathological analysis after the experiment. HE staining revealed well-organized hepatic cord and intact hepatic lobular in the control group, with a little bit interstitial collagen deposition and absence of hepatocyte degeneration or necrosis. In contrast, in the model group, the structure of hepatic cord was disorganized and the hepatic lobular was disrupted, with visible inflammatory cell infiltration and abnormal proliferation of pathologic blood vessels (Fig. 1F, H; *P* < *0.001*). In the model group, Masson staining demonstrated a mass of collagen deposition in liver tissue, compartmentalizing hepatocytes into clumps with distinct pseudo-lobe structures (Fig. 1F). Quantitative evaluation confirmed a sevenfold upregulation of collagen expression in the model group versus controls (Fig. 1F, I; *P* < *0.001*). Although there was still infiltration of inflammatory cells in the Rgkl-H group, the deposition of collagen was significantly reduced. (*P* < *0.001*). As a hallmark biomarker of HSC activation, α-SMA exhibited pronounced upregulation in the model group (Fig. 1F, J; *P* < *0.001*), while the expression of which could be differentially inhibited by Rgkl and Fzhy (*P* < *0.05, P* < *0.01, P* < *0.01*). Alanine aminotransferase and aspartate aminotransferase are clinically recognized as a method for detecting hepatic cell damage. As a result, serum biochemical analyses were carried out and results showed that ALT and AST levels in model mice were significantly increased in the model group (Fig. 1G; *P* < *0.001*), but both could be dramatically reversed by Rgkl (*P* < *0.001*). Biomarker analysis of hepatic fibrogenesis revealed a threefold elevation in serum HA levels in liver cirrhotic mice compared to normal controls (Fig. 1K; *P* < *0.001*). Both low and high doses of Rgkl significantly attenuated HA overproduction, demonstrating comparable therapeutic efficacy (*P* < *0.001*).

The above results demonstrated that Rgkl was effective in ameliorating hepatic parenchymal necrosis and tissue fibrosis, decreasing portal pressure and improving hepatic blood flow status. These effects were associated with the inhibition of HSC activation.

### Effect of Rgkl on hepatic vascular lesions in cirrhotic mice

**Fig. 2:**
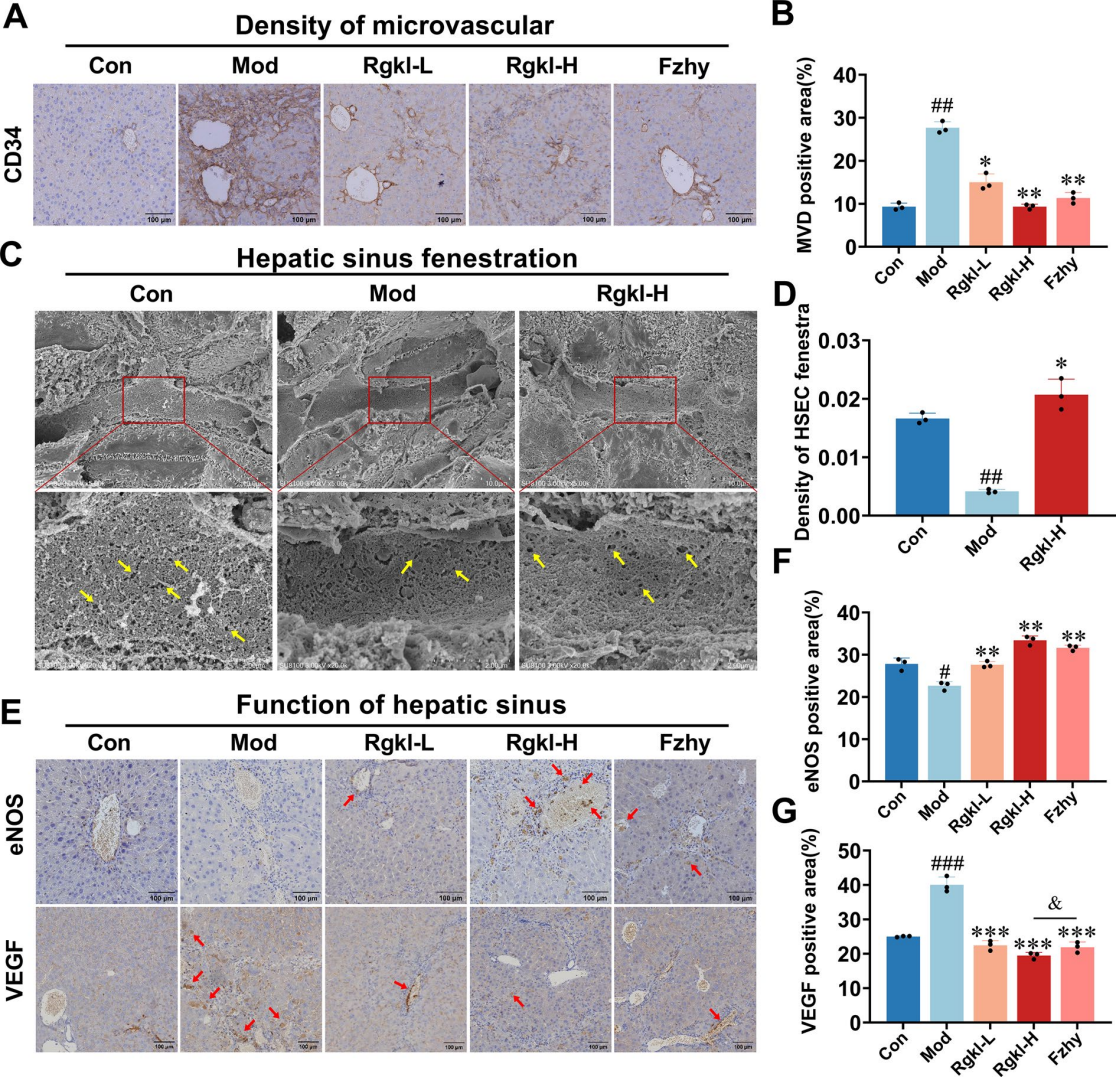
Effect of Rgkl on hepatic vascular lesions in cirrhotic mice. **A**, **B** Immunohistochemical detection of micro vessel density in liver tissue. **C**, **D** Observation of the structure and number of hepatic sinusoids’ fenestrae by scanning electron microscopy. **E**–**G** Immunohistochemical detection of eNOS and VEGF expression in LSEC. Data are presented as mean ± SD (3 mice per group). ^*#*^*P* < *0.05, *^*##*^*P* < *0.01, *^*####*^*P* < *0.001* compared with the control group; ^*^P < 0.05; ^**^P < 0.01, ^***^P < *0.001* compared with the model group; ^*&*^*P* < *0.05* compared with the Fzhy group

Micro vessel density (MVD) served as an indicator of micro vessel quantity, with CD34 commonly used to assess changes in micro vessel numbers. Immunohistochemical analysis demonstrated scant expression of CD34 in normal hepatic tissue, whereas cirrhotic models exhibited significant proliferation (Fig. 2A, B; *P* < *0.01*). Rgkl dose-dependently attenuated CD34-positive endothelial expansion (*P* < *0.05*; *P* < *0.01*), with the efficacy of high-dose paralleling the positive control Fzhy. These findings align with Rgkl's previously demonstrated hemodynamic improvements in hepatic microcirculation.

As specialized capillaries, the number of hepatic sinusoids’ fenestrae plays a key role in material exchange. After observations under scanning electron microscopy, the findings indicated a significant reduction in the number of fenestrae in the model group's liver sinusoids (Fig. 2C, D; *P* < *0.01*) while a steep increase after Rgkl high-dose treatment (*P* < *0.05*). Mechanistically, vascular mediators eNOS and VEGF are pivotal in maintaining sinusoidal fenestration. The model group displayed suppressed eNOS activity alongside a twofold upregulation of VEGF (Fig. 2E–G; *P* < *0.05*, *P* < *0.001*). Both low and high doses of Rgkl were able to increase eNOS levels (*P* < *0.01*) and exerted potent suppression of VEGF overexpression (*P* < *0.001*), with high-dose efficacy even surpassing Fzhy (*P* < *0.05*).

The results substantiate Rgkl's dual regulatory capacity to modulate the activity of eNOS and the expression of VEGF, thereby mitigating sinusoidal capillarization and pathological angiogenesis.

### Prediction of Rgkl’s pharmacodynamic composition for the treatment of liver cirrhosis

**Fig. 3:**
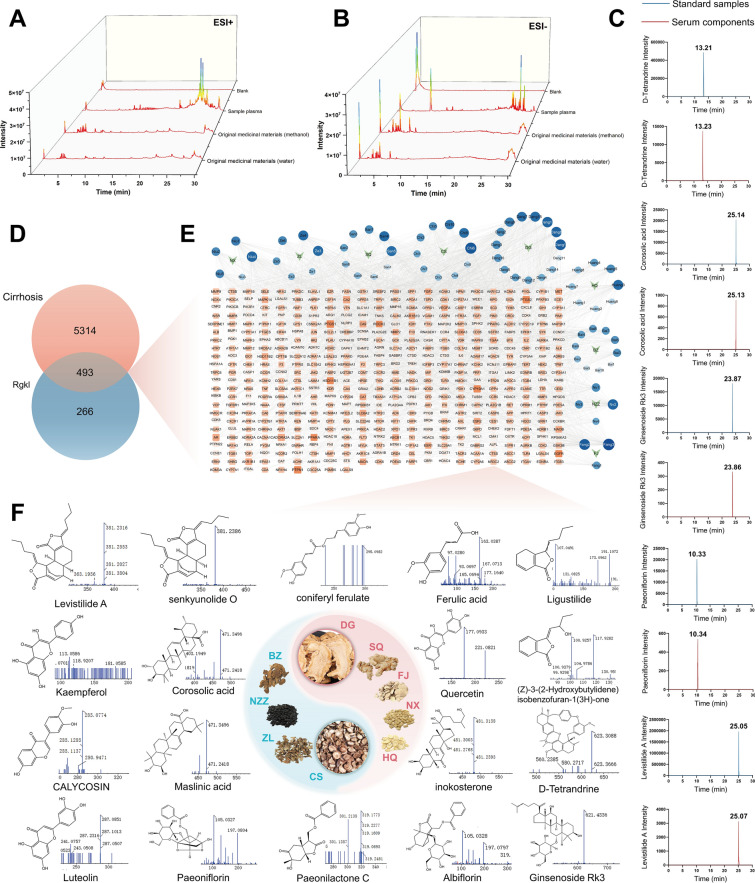
Prediction of Rgkl’s pharmacodynamic composition for the treatment of liver cirrhosis. **A**, **B** Total Ion Chromatogram (TIC) of Rgkl raw herb and drug-containing serum in positive and negative ion mode. **C** Comparative analysis of representative compound standards and serum samples. **D** Venn diagram of Rgkl blood-absorbable components and liver cirrhotic targets. **E** Network diagram illustrating the interconnections between drug components and disease targets. **F** Compatibility diagram of Rgkl prescription and chemical structures with secondary fragments of the top 18 active ingredients identified in blood components

In the preliminary phase, we identified 182 prototypic compounds from Rgkl using UPLC-Q-TOF-MS/MS. Subsequent analysis of its blood-absorbable components yielded total ion chromatograms in both positive and negative ionization modes (Fig. 3A, B). Following deduplication and subtraction of blank serum components, 72 blood-absorbable components were screened, including monoterpene glycosides, alkaloids, phenolic acids, saponins and flavonoids (Table. S2). To validate accuracy, representative components of selected drugs were chosen to analyzed. D-Tetrandrine (from *Stephania tetrandra*), Levistilide A (from *Ligusticum chuanxiong*), Corosolic acid (from *Eupatorium japonicum*), Ginsenoside Rk3 (from *Panax notoginseng*) and Paeoniflorin (from *Paeonia lactiflora*) exhibited the deviations of retention time within 0.1 min compared to their standards which confirms analytical precision. Further network pharmacology investigation collected 759 targets corresponding to Rgkl blood-absorbable components from TCMSP and Swiss Target Prediction databases. Continuing to intersect with 5,807 cirrhosis-related targets, 493 overlapping targets were identified (Fig. 3D). A network diagram that illustrated the interconnections between drug components and disease targets was then constructed (Fig. 3E), with top-ranked candidate bioactive components prioritized by degree values (chemical details in Table. S3; structures and MS/MS fragments in Fig. 3F). Notably, potential active ingredient of sovereign drugs DG and CS contributed approximately 50% of the prioritized candidates, including ligustilide, ferulic acid, paeoniflorin, Levistilide A and tetrandrine, suggesting their pivotal roles in improving liver cirrhotic by Rgkl.

### Prediction of Rgkl’s key targets and signaling pathway for the treatment of liver cirrhosis

**Fig. 4:**
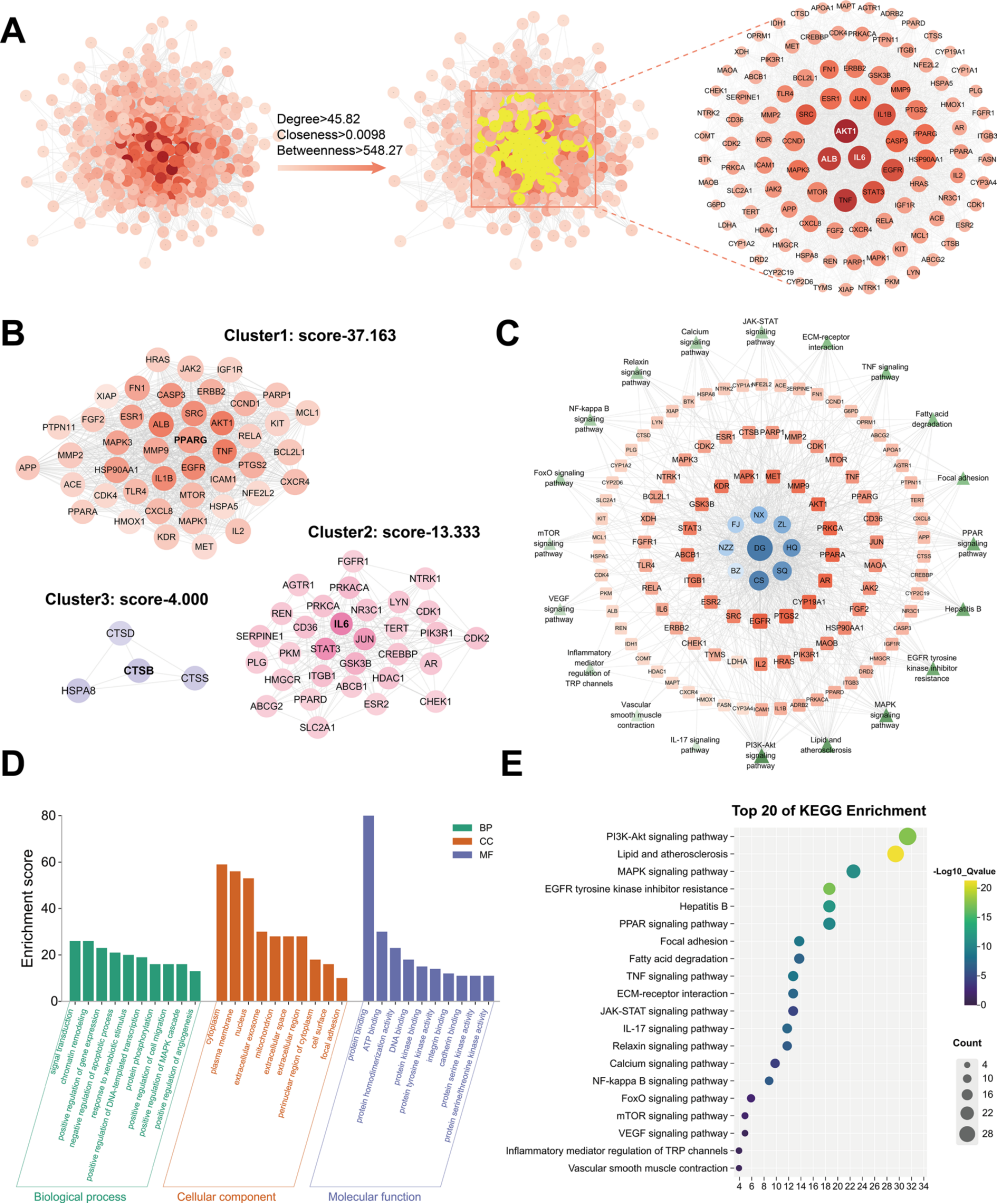
Network pharmacology identifies key targets of Rgkl in the treatment of liver cirrhosis. **A** PPI network for core targets screening. **B** Cluster analysis of core targets. **C** Network diagram linking drug, core targets, and pathways. **D** GO enrichment analysis of core targets. **E** KEGG enrichment analysis of core target

PPI network analysis of the 493 drug-target identified 102 core targets (Fig. 4A), which were subsequently stratified into three functional clusters through protein clustering analysis (Fig. 4B). Cluster 1, centered on PPARG, was linked to lipid metabolism, cell migration, cell proliferation and angiogenesis (Fig. S1). Cluster 2, based on IL6 protein, regulated inflammatory, immune responses, and metabolic homeostasis (Fig. S2). Cluster 3, focused on CTSB protein, was associated with immune defense processes (Fig. S3).

GO and KEGG enrichment analysis on 102 core targets identified 294 GO entries based on FDR. 193 of these related to biological processes were associated with signal transduction, angiogenesis regulation and protein phosphorylation; 38 of these related to cellular components were involved in focal adhesions, extracellular compartments, and mitochondria; and the remaining 63 were pertinent to molecular functions encompassing protein binding, integrin binding and Protein serine/threonine/tyrosine kinase activities and so on (Fig. 4D). KEGG pathway analysis demonstrated the multi-pathway regulatory capacity of Rgkl, including 20 signaling pathways like PI3K-Akt signaling, Fatty acid degradation, PPAR signaling, and ECM-receptor interactions (Fig. 4E). The component-core gene-pathway network (Fig. 4C) further highlighted the predominant therapeutic contributions of herbs like DG and CS.

### Transcriptomic detection of Rgkl’s effect on gene expression in liver tissue of cirrhotic mice

**Fig. 5:**
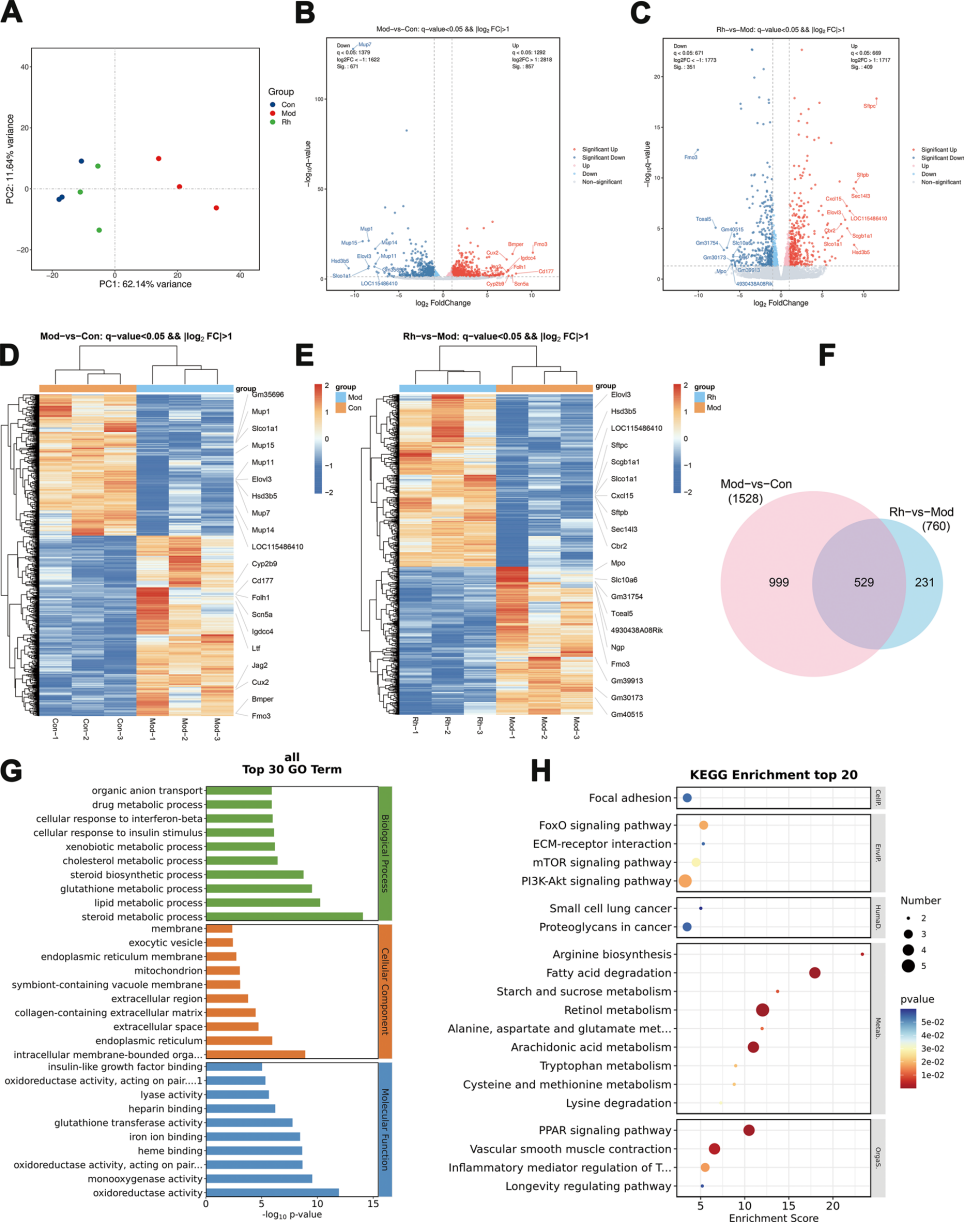
The expression of cirrhosis-related genes regulating by Rgkl. **A** PCA mapping of samples from Con, Mod, and Rh groups. **B**, **C** Volcano plots of Con vs Mod group and Mod vs Rh group. **D**, **E** Heat maps of DEGs expression profiles. **F** Venn diagram of DEGs’ intersection among the three groups. **G**, **H** GO and KEGG enrichment analysis

To elucidate changes in the transcriptome during liver cirrhotic progression and the mechanism of Rgkl's effect on portal hypertension, transcriptomic analysis was conducted on total RNA from liver tissues of Control (Con), Model (Mod), and Rgkl high dose (Rh) groups. The PCA plot revealed significant transcriptional differences among the groups, with Mod group exhibiting a rightward shift along the principal component compared to Con group and Rh group similar to control group (Fig. 5A).

Differential expressed genes (DEGs) analysis identified 1,528 DEGs with 857 upregulated and 671 downregulated in Con vs Mod comparisons while 760 DEGs with 409 upregulated and 351 downregulated in Rh vs Mod comparisons (Fig. 5B, C). Meanwhile, the heatmap showed pronounced differences in DEGs between each pair of groups (Fig. 5D, E). The intersection of Con vs Mod and Rh vs Mod DEGs (529 genes in total) was taken as target genes for exploring the role of Rgkl in the treatment of liver cirrhosis (Fig. 5F). The above intersection was subjected to GO, KEGG analysis (Fig. 5G, H), focusing on the 20 signaling pathways with the highest enrichment. Rgkl was found to treat liver cirrhosis mainly by affecting the signaling pathways such as PI3K-Akt signaling pathway, Fatty acid degradation, PPAR signaling pathway, and Vascular smooth muscle contraction.

**Fig. 6:**
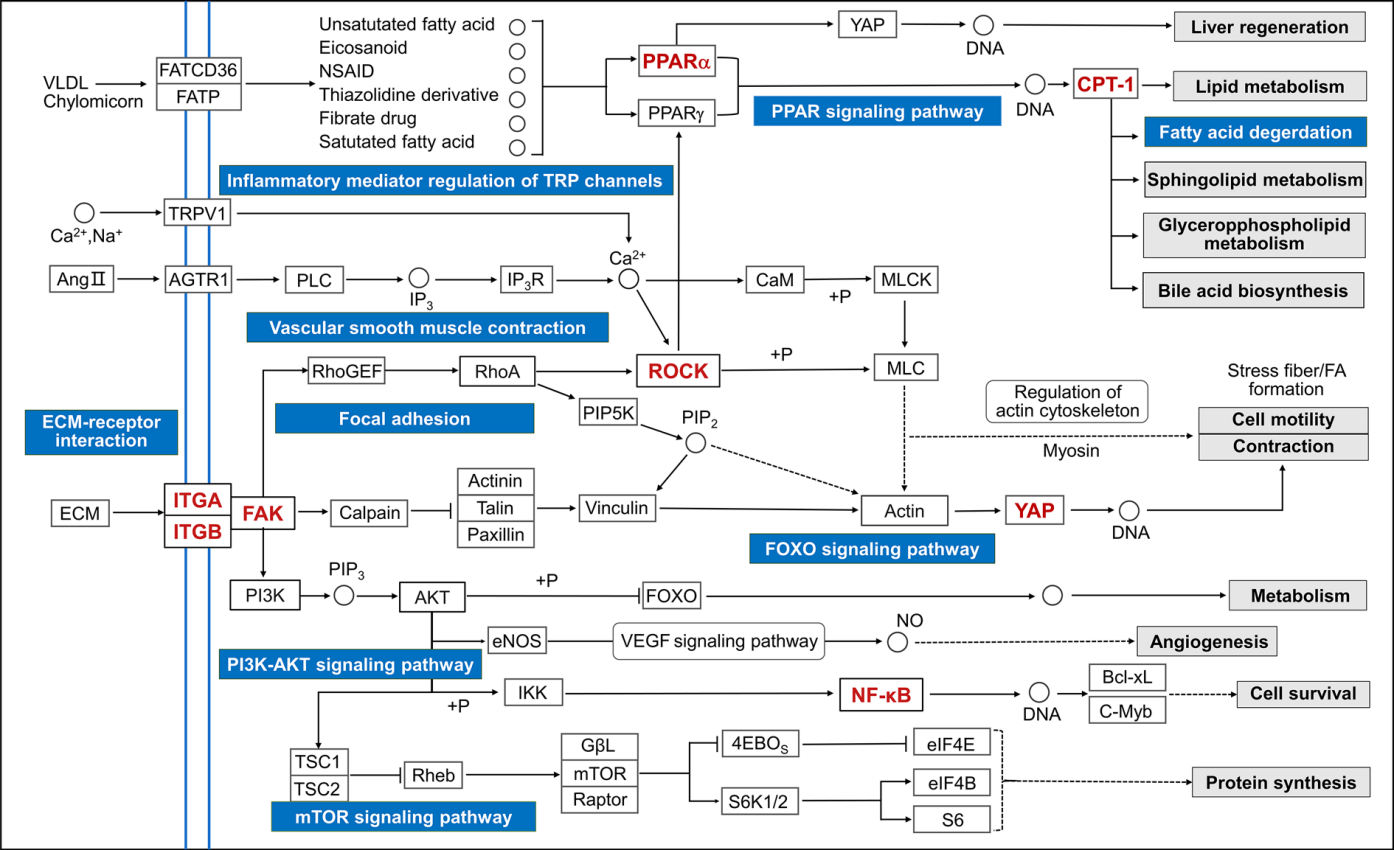
Network diagram of the mechanism of Rgkl for treating liver cirrhosis

The Top20 pathways obtained above were further intersected with the network pharmacology-enriched pathways to obtain a total of 9 pathways: PI3K-AKT signaling, FoxO signaling, mTOR signaling, ECM-receptor interaction, focal adhesion, vascular smooth muscle contraction, fatty acid degradation, PPAR signaling, and inflammatory mediator regulation of TRP channels. Using KEGG pathway mapping, we drafted a mechanistic network delineating Rgkl's anti-cirrhotic actions, linking upstream–downstream relationships among the nine signaling pathways mentioned above (Fig. 6). The network was functionally stratified into three core modules: lipid metabolism, mechanical mechanics and inflammatory activation, aligning with the three protein clusters identified in prior network pharmacology analyses.

Specifically speaking, Sphingolipid metabolism and Lipid metabolism signaling pathways mediated by CD36/PPAR/CPT-1 were associated with metabolic regulatory effects; RhoA/ROCK and YAP signaling pathways due to ECM/Focal adhesion kinase (FAK) were pertinent to intracellular mechanical mechanics regulation; and inflammatory activation was enriched to the more classical PI3K/AKT/NF-κB signaling pathway.

### Single-cell sequencing to study the primary target cells of Rgkl for the treatment of liver cirrhosis

**Fig. 7:**
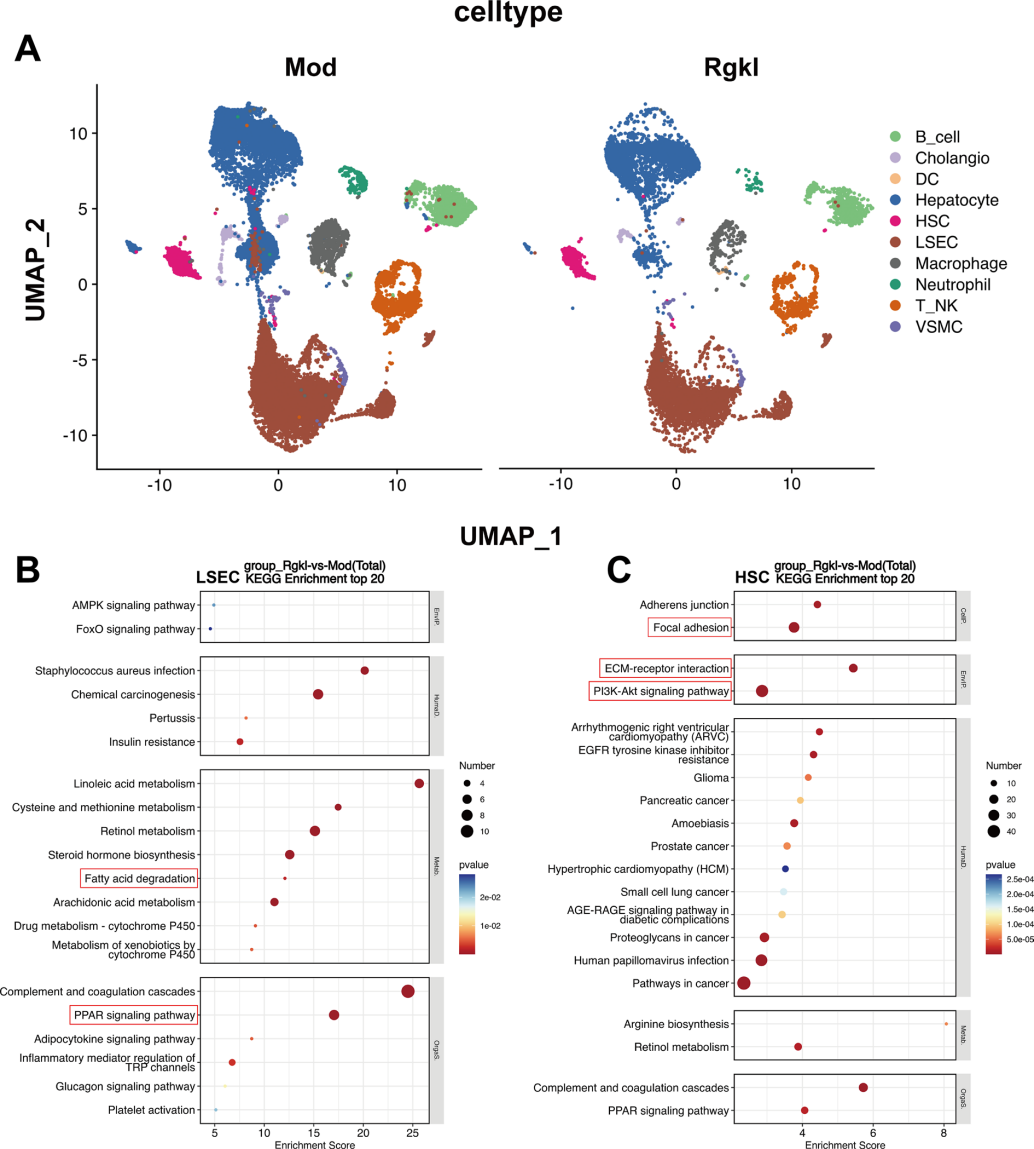
Single-cell sequencing affiliates transcriptome-enriched core pathways to different cells. **A** Celltype efficiently differentiates liver cells. **B**, **C** KEGG enrichment analysis of Top20 pathways within LSEC and HSC

As previously established, LSECs and HSCs are recognized as pivotal effector cells driving cirrhotic progression. While transcriptomic analysis has accurately identified core signaling pathways modulated by Rgkl, its specific effects on LSECs and HSCs require further elucidation. Consequently, single-cell sequencing technology was employed to systematically distinguish hepatic cell populations, leading to the identification of 10 distinct cell types, including LSECs and HSCs (Fig. 7A). Through KEGG pathway analysis of Rh vs Mod differentially expressed genes, we continued to analyze the effects of Rgkl on these two types of cells. Surprisingly, PPAR signaling pathway and fatty acid degradation, which midate sphingolipid and lipid metabolism, were found to be predominantly enriched in LSECs (with lower enrichment rankings in HSCs) (Fig. 7B). Additionally, Focal adhesion, ECM-receptor interaction associated with activation of mechanical mechanics and inflammatory pathway PI3K-Akt signaling pathway were also enriched in HSCs (Fig. 7C). These findings suggest that Rgkl exerts dual therapeutic effects by coordinately targeting LSEC and HSC functionalities in liver cirrhosis management.

### Validation experiments of Rgkl modulation of HSC and LSEC functions for treating liver cirrhosis

**Fig. 8:**
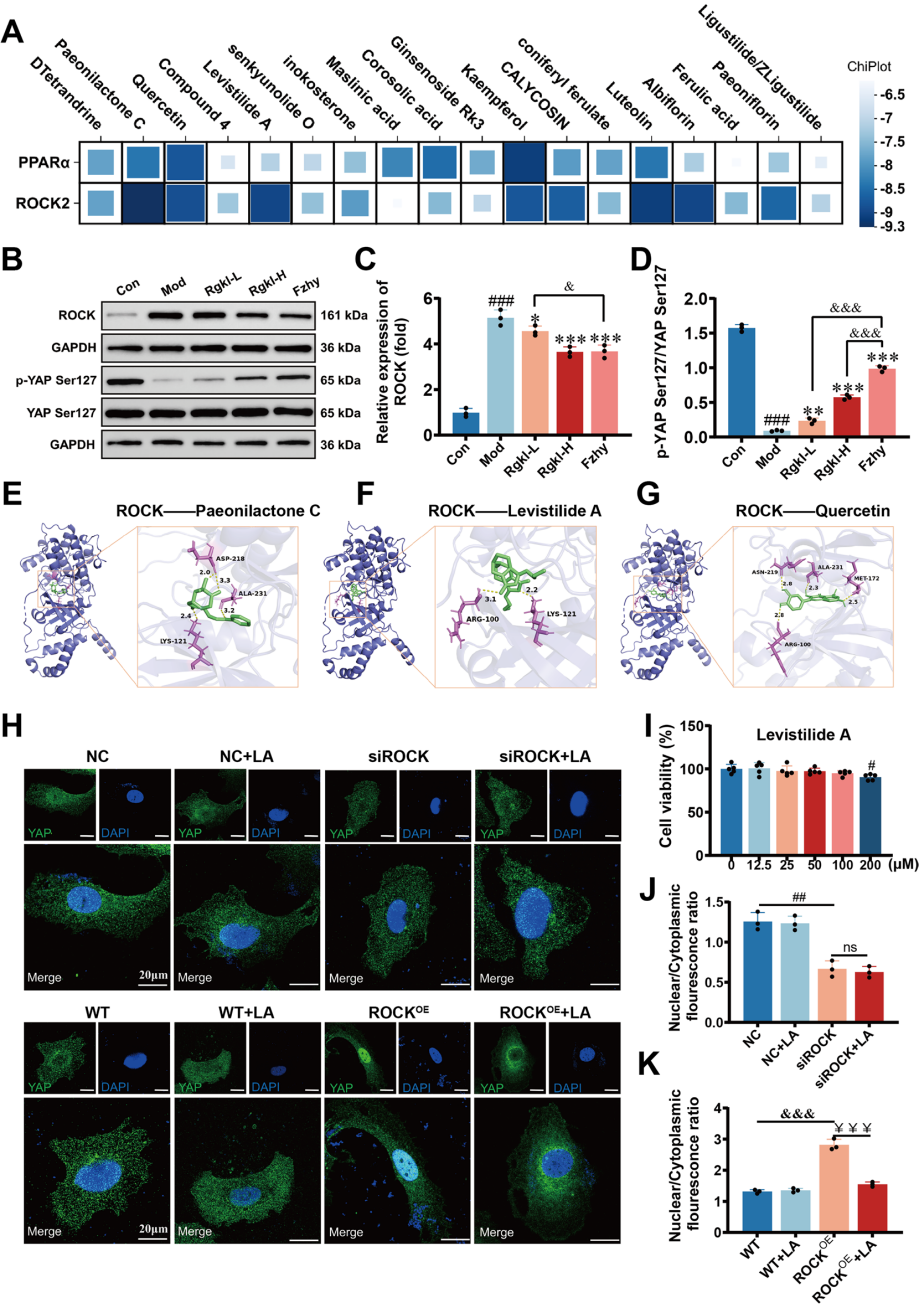
Validation experiments of Rgkl modulation of HSC mechanical activation for treating liver cirrhosis. **A** Heat maps showing the binding energies of PPARα and ROCK docked to some of the blood-absorbable components. Compound 4 is the component (Z)-3-(2-Hydroxybutylidene)isobenzofuran-1(3H)-one; **B**–**D** WB detection of protein levels of ROCK and YAP Ser127 site phosphorylation levels; (**E**–**G**) Visualization of ROCK molecular docking results; (**H**) Detection of YAP nuclear translocation by immunofluorescence; (**I**) CCK-8 detection of the maximum non-toxic dose of Levistilide A; (**J**, **K**) Quantitative analysis of YAP nuclear-to-plasmic ratio. Data are presented as mean ± SD (3 mice per group). ^*#*^*P* < *0.05, *^*##*^*P* < *0.001, *^*###*^*P* < 0.001; ^*^P < 0.05; ^**^P < 0.01, ^***^P < *0.001; *^*&&&*^*P* < *0.001*

To validate the single-cell sequencing findings, WB was performed to assess changes of key targets within core pathways of primary HSCs and LSECs from cirrhotic mice. In HSCs, the expression of key target ROCK, a critical mediator of ECM-receptor interaction and Focal adhesion, was observed to increase in cirrhotic models (Fig. 8B, C; *P* < *0.001*), which could be dose-dependently suppressed by Rgkl treatment (*P* < 0.05, *P* < *0.001*). Similarly, the mechanosensitive factor YAP downstream of ROCK exhibited marked dephosphorylation in liver cirrhotic HSCs (Fig. 8B, D; *P* < *0.001*). Both low and high doses of Rgkl reversed this phenomenon (*P* < *0.01*, *P* < *0.001*), though superior efficacy was noted for the positive control medicine Fzhy (*P* < *0.001*).

**Fig. 9:**
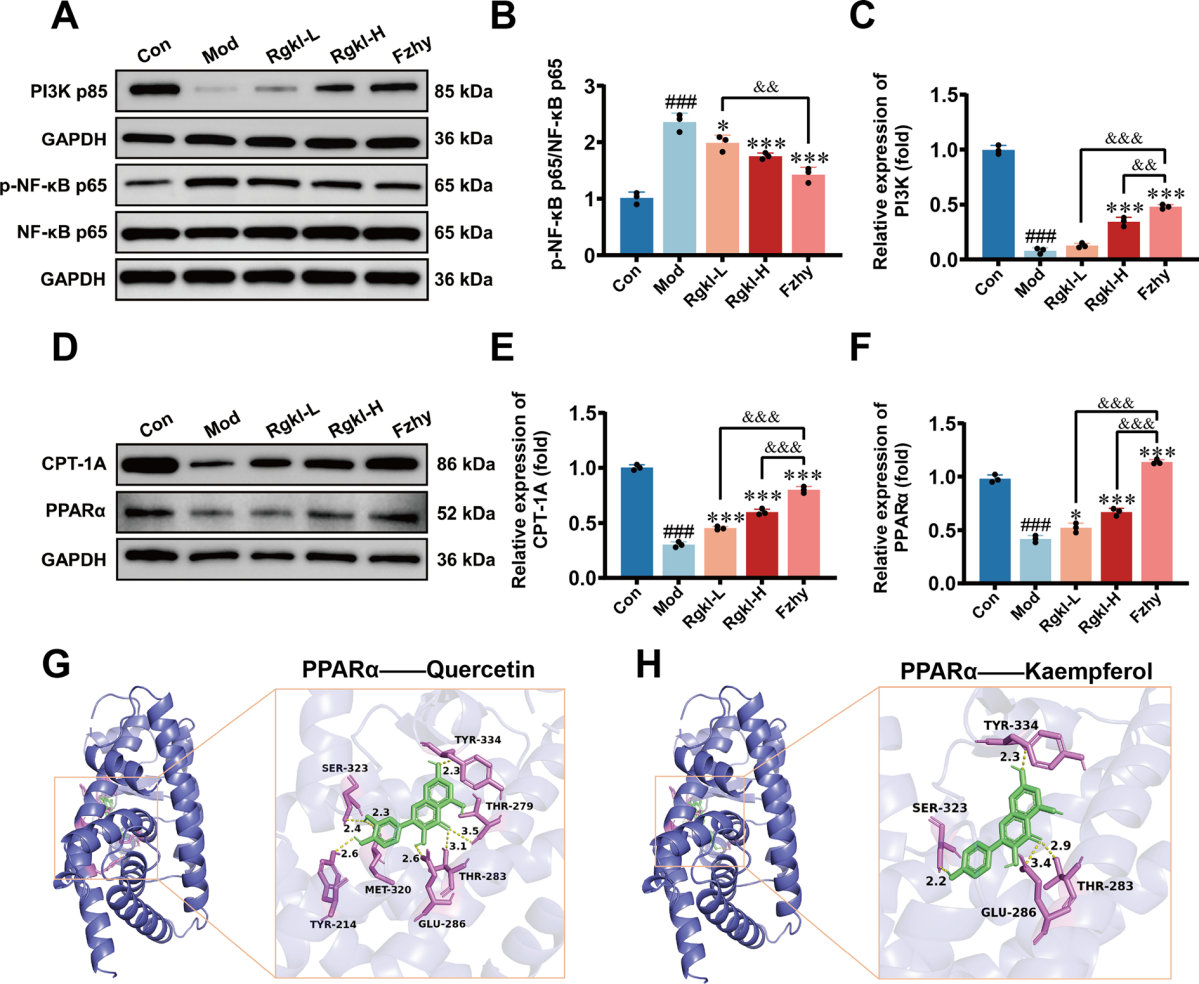
Validation experiments of Rgkl modulation of HSC inflammatory activation and LSEC lipid metabolism for treating liver cirrhosis. **A**–**C** WB detection of NF-κB phosphorylation levels and protein levels of PI3K; (**D**–**F**) WB detection of protein levels of CPT-1A and PPARα expression; (**G**–**H**) Visualization of PPARα molecular docking results

Basically, to screen the pharmacologically active ingredients targeting ROCK, molecular docking was employed to find that Paeonilactone C, Quercetin, Levistilide A, and Luteolin exhibited high binding energy with ROCK (Fig. 8A, E–G). Next, Levistilide A was chosen as a representative for validation. As a result, immunofluorescence revealed no significant change in the nuclear localization of YAP in NC and ROCK-knockdown HSCs (Fig. 8H, J), but showed significant inhibition of nuclear translocation in ROCK-OE HSCs (Fig. 8H, K; *P* < *0.001*), confirming its role as a mechanical activation component of Rgkl in improving cirrhotic portal hypertension. Concurrent analysis demonstrated suppression of inflammatory activation in HSCs by low and high doses of Rgkl (Fig. 9A, B; *P* < *0.05*).

In primary LSECs, PPARα/CPT-1A involved in lipid oxidative catabolism was downregulated in liver cirrhotic mice (Fig. 9C–E; *P* < *0.001*), which was able to be differentially reversed by low and high doses of Rgkl (*P* < *0.001*). Molecular docking performed on PPARα identified pharmacodynamic components of Rgkl like Quercetin and Kaempferol may be the key to ameliorate liver cirrhosis by modulating the lipid metabolism in LSECs (Fig. 9F–G). Therefore, we conclude that Rgkl may ameliorate liver cirrhosis from three aspects of lipid metabolism, mechanical and inflammatory activation, as well as the two cells of HSC and LSEC.

## Discussion

A growing body of evidence suggests that a synergistic interaction among LSEC endothelial dysfunction, capillarization, and HSC myofibroblastic transition, together with their reciprocal positive feedback loops, plays a pivotal role in driving continuous progression of liver cirrhosis. Firstly, LSEC dominates the dysregulated expression of vasoactive mediators such as ET-1/NO and TXA2/NO [13], which not only induces mechanical activation of HSC but also stimulates ECM deposition, consequently remodeling the biomechanical microenvironment of hepatic sinusoids [14]. ECM components from HSC, including FN and COL1A1, reciprocally modulate the phenotypic characteristics and functional behavior of LSEC through integrin receptors [15]. This study employed a network pharmacology approach to preliminarily identify several disease targets associated with liver cirrhosis. Subsequent transcriptomic sequencing analysis revealed the twenty most significantly enriched pathways related to liver cirrhosis, encompassing mechanical activation, inflammatory response, metabolic regulation, and immune modulation. We constructed a comprehensive network diagram to visually demonstrate the multi-pathway synergistic therapeutic effects of Rgkl in liver cirrhosis treatment. To precisely delineate the cellular and molecular targets of the drug, we categorized the key pathways and their associated cells through integrating single-cell sequencing analysis. Our findings demonstrate that ECM mediates activation of the Integrin/FAK mechanical signaling pathway. This pathway not only modulates cellular metabolism, survival, and tissue inflammation through regulating PI3K-AKT signaling [16], but also activates RhoA/ROCK signaling to facilitate cytoskeletal remodeling. The latter promotes YAP nuclear translocation, leading to the activation of fibrosis-promoting transcription factors upon interaction, thereby establishing a mechano-biochemical coupling feedback loop. These mechanisms were conclusively validated through a systematic investigation into the mechanical signaling pathway activation of HSC [3]. The network analysis further revealed the involvement of the PPAR signaling pathway in liver cirrhotic pathogenesis, a pathway frequently dysregulated in chronic liver diseases induced by alcohol exposure, high-fat diets, and insulin resistance. Notably, significant enrichment of PPAR signaling was observed specifically in LSEC. We hypothesize that the release of the cancer-related active ingredient S1P by LSEC necessitates inhibition of the key molecules PPARα and CPT-1A under mechanical stress, acting as a protective measure to fulfill sphingolipid biosynthesis demands. Consequently, current therapeutic strategies predominantly employ PPAR agonists to alleviate intercellular lipid accumulation, thereby improving IHVR and ameliorating fibrotic progression [17, 18]. These findings indicate that distinct cell populations contribute synergistically via multiple pathways and mechanisms during the progression and exacerbation of liver cirrhosis.

In traditional Chinese medicine research, elucidating pharmacological efficacy necessitates identifying credible medicinal ingredients as a fundamental prerequisite. Having established the therapeutic effects of Rgkl on liver cirrhosis, we first employed UPLC-Q-TOF-MS/MS to systematically profile its bioavailable components, which constitute the pharmacodynamic material basis. Subsequently, leveraging transcriptomic sequencing and network pharmacology databases, we ranked the therapeutic potential of these pharmacodynamic components by prioritizing targets identified for liver cirrhosis modulation. Target validation was then conducted through molecular docking, Western blotting, and immunofluorescence assays, specifically focusing on targets affiliated with particular cell populations identified by single-cell sequencing. For pharmacological validation, we rigorously monitored core intracellular pathways enriched by network pharmacology analysis. Primary HSC isolated from cirrhotic mice were subjected to systematic evaluation of ECM-receptor interactions and focal adhesion machinery, examining the expression of ROCK and the phosphorylation status of YAP. Strikingly, both low and high doses of Rgkl administration effectively suppressed ROCK expression and YAP phosphorylation, achieving concomitant inhibition of HSC hypercontractility and overexpression of fibrotic markers. Molecular docking analysis identified active ingredients such as Paeonilactone C and Levistilide A as strongly binding to ROCK, suggesting their potential for mechanically mediated blockade. Focusing on Levistilide A as a representative compound, we conducted genetic perturbation experiments using HSC with varying ROCK expression levels to detect YAP nuclear translocation in the presence or absence of Levistilide A. This demonstrates that Levistilide A is a pharmacologically relevant component targeting ROCK, effectively explaining the remarkable efficacy of Rgkl in enhancing hepatic sinusoidal blood flow. Extending this paradigm, we further investigated Rgkl's modulatory effects on the inflammatory responses of HSC. Both low- and high-dose Rgkl administration significantly suppressed the phosphorylation of NF-κB downstream of PI3K/AKT, effectively inhibiting HSC activation and ECM deposition and thereby attenuating the progression of fibrosis [9, 19]. Emerging evidence indicates that hyperactivation of the PI3K/AKT pathway in hepatocytes causes vascular malformations, a mechanism pharmacologically verified using the AKT inhibitor miransertib [20]. These findings are consistent with our observations of tissue fibrosis and reduced pathological angiogenesis. However, whether Rgkl elicits comparable therapeutic outcomes requires further comprehensive mechanistic validation. In primary LSEC, both low and high doses of Rgkl treatment upregulated the expression of PPARα and CPT-1A, concomitantly enhancing lipid oxidative capacity. This effect could contribute to suppressing S1P overproduction, thus potentially reducing the risk of carcinogenesis. Through molecular docking, we preliminarily screened several pharmacodynamic components including quercetin and kaempferol, which may target PPARα. It is well recognized that lipid accumulation in HC promotes the synthesis of NADP and cholesteryl esters, leading to inactivation of the tumor suppressor PTEN and an elevated risk of carcinogenesis [21]. Our study further highlights the hazards of lipid accumulation in LSEC. The screened compounds demonstrate potential for synergistic application with PPARα agonists like fenofibrate to reduce the risk associated with hepatic lipid exposure [22]. Nevertheless, this remains speculative until more rigorous experiments confirm the binding affinity of targeted pharmacodynamic ingredients.

In summary, this study elucidated the intrinsic mechanism of Rgkl in improving liver cirrhosis from multiple dimensions, including biomechanical regulation, inflammation, and lipid metabolism. The dominant role of mechanical factors in chronic liver disease represents an emerging research direction and a notable highlight of this study. Nevertheless, limitations remain, such as whether the identified pathways universally apply to liver diseases with different etiologies, and whether the comprehensive evaluation of the five components of traditional Chinese medicine research (biosynthetic components, original components, transformed components, prototype components, and effector components) has been adequately addressed, warranting further investigation.

## Conclusion

This study established a synergistic framework integrating bioinformatic virtual prediction with transcriptomic profiling and single-cell sequencing validation to elucidate the molecular mechanism of Rgkl for the treatment of liver cirrhosis. Meanwhile, it systematically mapped core pathways to specific hepatic cell populations (involving multiple dimensions of mechanical mechanics, inflammation, and lipid metabolism). Upon this foundation, this study identified the potential of components such as Levistilide A and Quercetin to treat liver cirrhosis by intervening in the contraction of HSC and the lipid metabolism of LSEC.

## Supplementary Information

Supplementary Material 1

## Data Availability

No datasets were generated or analysed during the current study.

## Abbreviations

DEGs: Differential expression gene
ET-1: Endothelin-1
ECM: Extracellular matrix
HSCs: Hepatic stellate cells
IHVR: Intrahepatic vascular resistance
LSECs: Liver sinusoidal endothelial cells
MVD: Micro vessel density
PH: Portal hypertension
PPI: Protein‒protein interaction
Rgkl: Rou gan keli
S1P: Sphingosine-1-phosphate
TCM: Traditional Chinese Medicine
VEGF: Vascular endothelial growth factor
WB: Western blot
