# Supplementary material for: Integration of network pharmacology, transcriptomics and single-cell sequencing to explore the effect of Rougan Keli in alleviating liver cirrhosis
Source: Chin Med. 2025 Oct 2;20:153. doi: 10.1186/s13020-025-01220-z (PMC12490168; doi:10.1186/s13020-025-01220-z)

Supplementary data

Table. S1 Combination dosage of Rgkl

No.	Chinese name	Latin name	Vegetal product	Family	Part used	Dose (g)
1	Danggui (DG)	Angelica sinensis (Oliv.) Diels	Angelicae Sinensis Radix	Apiaceae	Root	168
2	Chishao (CS)	Paeonia lactiflora Pall.	*Paeoniae* Radix Rubra	Ranunculaceae	Root	252
3	Sanqi (SQ)	*Panax notoginseng* (Burk.) F.H.Chen	*Notoginseng* Radix et Rhizoma	Araliaceae	Rhizome	101
4	Zelan (ZL)	*Lycopus lucidus* Turcz.var.hirtus Regel	*Lycopi* Herba	Asteraceae	Aboveground	168
5	Niuxi (NX)	Achyranthes bidentata Bl.	Achyranthis Bidentatae Radix	Amaranthaceae	Root	168
6	Nvzhenzi (NZZ)	Ligustrum lucidum Ait.	*Ligustri* Fructus	Oleaceae	Fruit	252
7	Huangqi (HQ)	Astragalus membranaceus (Fisch.) Bge.	Astragali Radix	Fabaceae	Root	252
8	Baizhu (BZ)	Atractylodes macrocephala Koidz.	Atractylodis Macrocephalae Rhizoma	Asteraceae	Rhizome	168
9	Fangji (FJ)	Stephania tetrandra S.Moore	Stephaniae Tetrandrae Radix	Menispermaceae	Root	101

Table S2 Identification results of the blood components of Rgkl based on UPLC-Q-TOF-MS/MS

No.	t_R/min	Compound name	Molecular formula	M(Da)	Ion type	Error(ppm)	Characteristic Fragment	Source
1	13.03	Ferulic acid	$C_{10}H_{10}O_4$	194.1800	$[M+H]^+$	-0.2	163.0393, 177.1618<br>97.0282	A
2	9.85	Anisic acid	$C_8H_8O_3$	152.0473	$[M+H]^+$	-1.6	153.1274, 97.0649<br>69.0697, 77.0383<br>107.0849	A
3	3.13	Cinnamic acid	$C_9H_8O_2$	148.0524	$[M+H]^+$	-9.6	149.0241, 103.0543<br>77.0387, 121.0304<br>79.0543, 51.0228	A
4	25.03	Senkyunolide A	$C_{12}H_{16}O_2$	192.2600	$[M+H]^+$	1	193.1567, 79.0535<br>81.0326, 149.0204	A
5	18.28	Butylphathlide	$C_{12}H_{14}O_2$	190.2400	$[M+H]^+$	-1	107.0485, 77.0387<br>191.1078, 79.0532<br>145.1011	A
6	20.68	Osthole	$C_{15}H_{16}O_3$	244.2900	$[M+H]^+$	-9.1	245.1154, 245.1360<br>157.1000, 227.1789	A
7	18.28	Ligustilide/Z-Ligustilide	$C_{12}H_{14}O_2$	190.2400	$[M+H]^+$	-0.6	107.0491, 77.0373<br>191.1072, 79.0528<br>173.0962	A
8	24.62	Levistilide A	$C_{24}H_{28}O_4$	380.4800	$[M+H]^+$	-2.3	381.2188, 381.2602<br>381.2514, 363.1922	A
9	9.83	coniferyl ferulate	$C_{20}H_{20}O_6$	355.1190	$[M-H]^-$	0.8	295.0982, 159.0642	A
10	25.03	3-N-butyl-4,5-dihydrophthalide	$C_{12}H_{16}O_2$	192.2540	$[M+H]^+$	1	193.1567, 79.0535<br>81.0326, 149.0204	A

Continued Table S2

11	24.62	senkyunolide O	$C_{24}H_{28}O_4$	380.4800	$[M+H]^+$	-2.3	381.2386	A
12	24.15	Senkyunolide H	$C_{12}H_{16}O_4$	224.1049	$[M+H]^+$	-8.6	225.1025, 67.0529	
							225.1486, 207.0804	A
							77.0366, 181.0750	
13	19.87	4-Hydroxy-3-butylphthalide	$C_{12}H_{14}O_3$	206.2400	$[M+H]^+$	-2.3	207.0861, 91.0522	
							119.0158, 163.0390	A
							95.0838, 161.0920	
14	24.15	Senkyunolide I	$C_{12}H_{16}O_4$	224.1049	$[M+H]^+$	-8.6	225.1394, 67.0529	
							77.0366, 181.0750	A
							91.0548, 209.0257	
15	15.68	N-butylidenephthalide	$C_{12}H_{12}O_2$	188.0837	$[M+H]^+$	-5.4	161.0922, 115.0523	
							189.0913, 102.9563	A
							77.0372, 171.0803	
16	22.97	(Z)-3-(2-Hydroxybutylidene)is obenzofuran-1(3H)-one	$C_{12}H_{12}O_3$	204.2200	$[M-H]^-$	-2.6	100.9258, 73.2164	
							100.9353, 58.9722	A
							104.9764, 116.9267	
17	4.72	Paeoniflorin	$C_{23}H_{28}O_{11}$	481.1704	$[M+H]^+$	-0.1	105.0327, 197.0804	
							133.0637, 151.0739	B
							179.0708, 319.1171	
18	4.77	Albiflorin	$C_{23}H_{28}O_{11}$	481.1702	$[M+H]^+$	-0.4	197.0797, 133.0631	B
							151.0746, 105.0328	
19	23.93	Paeonilactone C	$C_{17}H_{18}O_6$	318.3200	$[M+H]^+$	9.60	319.1655, 301.1872	
							287.2012, 176.9942	B
							57.0683, 122.9724	

Continued Table S2

20	18.00	paeoniflorigenone	$C_{17}H_{18}O_6$	319.1152	$[M+H]^+$	9.6	319.1655, 79.0552<br>301.1481, 287.2012<br>57.0683, 122.9724	B
21	25.04	Queen Bee acid	$C_{10}H_{18}O_3$	186.2500	$[M+H]^+$	0.2	67.0539, 109.1009<br>55.0542, 155.1064<br>81.0695, 83.0837	B
22	24.36	oxypaeoniflora	$C_{23}H_{28}O_{12}$	496.4600	$[M+H]^+$	-9.50	497.3320, 425.2253<br>313.2676, 347.0169<br>210.9284, 151.0054	B
23	10.68	Vanillic acid	$C_8H_8O_4$	169.0491	$[M+H]^+$	-2.4	169.0739, 123.1163<br>107.0844, 113.0214<br>55.0551, 91.0533	B
24	9.94	mudanpioside E	$C_{24}H_{30}O_{13}$	525.1609	$[M-H]^-$	-0.9	121.0295, 165.0563<br>327.1083, 525.0259<br>449.1441, 119.0347	B
25	24.92	Hederagenin	$C_{30}H_{48}O_4$	472.7100	$[M-H]^-$	0.1	471.3506, 100.9327<br>99.0072, 453.3413<br>427.3609, 116.9268	B
26	7.60	(+)-paeonilactone B	$C_{10}H_{12}O_4$	196.2000	$[M-H]^-$	-1.7	160.8418, 96.9602<br>151.0765, 123.0435<br>195.0854, 179.0529	B
27	0.86	L-Glutamic acid	$C_5H_9NO_4$	146.0458	$[M-H]^-$	0	102.0564, 128.0352<br>128.0465, 146.0457	C, H
28	11.92	Kaempferol	$C_{15}H_{10}O_6$	287.0493	$[M+H]^+$	-19.6	113.0606, 111.1158<br>163.0710, 174.0983	C

Continued Table S2

29	28.37	Notoginsenoside T5	$C_{41}H_{68}O_{12}$	753.4694	$[M+H]^+$	-11.8	753.4782, 753.5128<br>753.5386, 753.4535	C
30	23.20	Ginsenoside Re	$C_{48}H_{82}O_{18}$	945.5417	$[M-H]^-$	-1.2	945.5371, 945.5724	C
31	23.20	Ginsenoside Rd	$C_{48}H_{82}O_{18}$	945.5417	$[M-H]^-$	-1.2	945.5371, 945.5724<br>945.5857,	C
32	28.20	Ginsenoside Rk3	$C_{36}H_{60}O_8$	621.4328	$[M+H]^+$	-5.2	621.4336	C
33	23.93	Falcarinol	$C_{17}H_{24}O$	245.1895	$[M+H]^+$	-1.7	245.1877, 161.1341<br>245.1375, 55.0524	C
34	23.20	GYPENOSIDE-XVII	$C_{48}H_{82}O_{18}$	945.5417	$[M-H]^-$	-1.2	945.5371, 945.5724<br>945.5857	C
35	28.41	Diisooctyl phthalate	$C_{24}H_{38}O_4$	391.2844	$[M+H]^+$	0.3	149.0231, 167.0335<br>57.0700, 71.0847	C
36	14.49	Quercetin	$C_{15}H_{10}O_7$	301.0383	$[M-H]^-$	9.8	177.0932	C, D, G, I
37	24.93	Corosolic acid	$C_{30}H_{48}O_4$	471.3467	$[M-H]^-$	-2.5	61.9883, 100.9327<br>471.3747, 453.3335	D
38	15.27	1-(2-Hydroxy-5-methylphenyl) ethanone	$C_9H_{10}O_2$	151.0752	$[M+H]^+$	-0.6	95.0486, 93.0695<br>91.0539, 109.0638	D
39	11.92	Luteolin	$C_{15}H_{10}O_6$	287.0493	$[M+H]^+$	-19.6	269.0398, 287.0523<br>287.1434, 111.1158	D
40	26.49	apigenin-7-O-β-D-glucopyrano side	$C_{21}H_{20}O_{10}$	431.1056	$[M-H]^-$	16.8	91.0216, 415.0706<br>431.3148	D
41	24.84	Thymol	$C_{10}H_{14}O$	151.1117	$[M+H]^+$	0.1	151.1118, 133.0927<br>95.0490, 81.0316	D

Continued Table S2

42	26.33	Ursolic acid acetate	$C_{32}H_{50}O_4$	499.3765	$[M+H]^+$	-3.2	499.3761	D, F
43	26.94	inokosterone	$C_{27}H_{44}O_7$	481.3159	$[M+H]^+$	-4.8	481.3138, 481.2922<br>481.2622, 481.338	E
44	11.81	Baicalin	$C_{21}H_{18}O_{11}$	447.0933	$[M+H]^+$	2.6	271.0601, 447.0932<br>447.7900, 73.0463<br>431.0876	E
45	16.31	Wogonin	$C_{16}H_{12}O_5$	285.0760	$[M+H]^+$	0.9	285.0756, 285.0991<br>169.0615, 155.0840<br>121.0976	E, G
46	0.88	Betaine	$C_5H_{11}NO_2$	118.0861	$[M+H]^+$	-0.9	58.0667, 59.0731<br>118.0919, 72.0851<br>57.0573	E
47	26.33	Oleanolic acid 3-acetate	$C_{32}H_{50}O_4$	499.3761	$[M+H]^+$	-3.2	499.3761	E, F
48	26.54	alpha-Spinasterol glucoside	$C_{35}H_{58}O_6$	573.4152	$[M-H]^-$	-1.5	59.0145, 573.4044<br>573.4186, 125.0613	E
49	13.95	Azelaic acid	$C_9H_{16}O_4$	187.0974	$[M-H]^-$	-0.6	125.0972, 187.0989<br>87.0666, 123.0818<br>169.0857	E
50	22.24	Caffeic acid	$C_9H_8O_4$	181.0496	$[M+H]^+$	0.4	181.1616, 139.0383<br>181.0472, 149.0228	F
51	10.29	olivetolic acid	$C_{12}H_{16}O_4$	225.1101	$[M+H]^+$	-9	225.1340, 209.0293<br>225.0865, 211.0095	F
52	21.75	Oleuropein	$C_{25}H_{32}O_{13}$	539.1809	$[M-H]^-$	7.3	539.1851, 152.9942<br>539.1628, 208.9344<br>385.1766	F

53	24.93	Maslinic acid	$C_{30}H_{48}O_4$	471.3467	$[M-H]^-$	-2.5	471.3465, 100.9327<br>471.3747, 112.985<br>453.3335	F
54	22.94	TILIROSIDE	$C_{30}H_{26}O_{13}$	593.1373	$[M-H]^-$	-0.1	121.0296, 593.1304<br>121.0379, 505.1128<br>525.1143	G
55	16.24	ISOMUCRONULATOL	$C_{17}H_{18}O_5$	303.1204	$[M+H]^+$	-7.5	303.1452, 303.1169<br>271.2647, 131.0837	G
56	16.30	Glycitein	$C_{16}H_{12}O_5$	285.0760	$[M+H]^+$	0.9	285.0756, 285.0991<br>169.0615, 79.0522	G
57	13.37	CALYCOSIN	$C_{16}H_{12}O_5$	285.0757	$[M+H]^+$	0.1	285.0750, 285.0892<br>285.1048, 185.0585	G
58	23.64	(6aR, 11 aR)-3-hydroxy-9,l0-diMethoxy pterocarpan--7--O--β--D--glucoside	$C_{23}H_{26}O_{10}$	463.1642	$[M+H]^+$	9.5	407.0996, 463.1674<br>463.1842, 463.2638<br>275.0078	G
59	20.55	Pratensein 7-O-glucopyranoside	$C_{22}H_{22}O_{11}$	461.1082	$[M-H]^-$	-1.4	121.0286, 417.0826<br>461.1103, 104.9257<br>163.0404	G
60	23.81	7,2'-dihydroxy-3',4'-dimethoxyisoflavane-7-O-glucoside	$C_{23}H_{28}O_{10}$	463.1571	$[M-H]^-$	-8.3	209.1176, 253.1086<br>135.0454, 209.1326	G

61	23.98	8beta-Methoxyatractylenolide I	$C_{16}H_{22}O_3$	261.1495	$[M-H]^-$	-0.3	217.1608	H
62	26.23	Icariside F2	$C_{18}H_{26}O_{10}$	401.1417	$[M-H]^-$	-9	116.9276, 116.9365<br>100.9329, 121.0282<br>112.9858	H
63	23.53	atractyloside B	$C_{21}H_{38}O_{10}$	449.2430	$[M-H]^-$	8.5	449.2441, 391.2861<br>313.2388	H
64	23.16	Atractylenolide II	$C_{15}H_{20}O_2$	233.1534	$[M+H]^+$	-0.7	233.1528, 217.1215	H
65	19.32	5-Hydroxymethylfurfural	$C_6H_6O_3$	127.0386	$[M+H]^+$	-2.6	53.0379, 99.0444<br>81.0336, 57.0334<br>55.017	H
66	17.97	Eudesma-4(15),7(11)-dien-8-on e	$C_{15}H_{22}O$	219.1740	$[M+H]^+$	-1.4	219.1727, 203.1400<br>219.1580, 121.0996<br>67.0555	H
67	0.88	Valine	$C_5H_{11}NO_2$	118.0861	$[M+H]^+$	-0.9	58.0667, 55.0549<br>59.0731, 118.0919<br>72.0851	H
68	0.91	D-Proline	$C_5H_9NO_2$	116.0705	$[M+H]^+$	-0.5	70.0715, 116.0745	H
69	19.33	Uridine	$C_9H_{12}N_2O_6$	245.0758	$[M+H]^+$	-4	245.0768, 245.0856<br>245.0998, 245.1174<br>115.0397	H
70	27.91	Stearic acid	$C_{18}H_{36}O_2$	285.2789	$[M+H]^+$	0.4	285.2747, 285.2864<br>57.0693, 285.247<br>89.059	H

Continued Table S2

71	6.34	D-Tetrandrine	$C_{38}H_{42}N_2O_6$	623.3122	$[M+H]^+$	1	623.3088, 594.3022<br>592.2617, 580.2717<br>192.0949, 176.1028	I
72	12.66	Cycleanine	$C_{38}H_{42}N_2O_6$	622.3042	$[M+H]^+$	0	623.3129	I

Note: A: *Angelica sinensis* (Oliv.) Diels; B: *Paeonia sinjiangensis* K. Y. Pan; C: *Panax pseudo-ginseng var. notoginseng* (Burkill) Hoo et Tseng; D: *Lycopus maackianus* (Maxim. ex Herder) V. Komarov; E: *Achyranthes bidentata* Blume; F: *Ligustrum lucidum* W. T. Aiton; G: *Astragalus membranaceus* Moench; H: *Atractylodes macrocephala* Koidz.; I: *Sinomenium acutum* (Thunb.) Rehder & E. H. Wilson.

Table. S3 The top 18 active ingredients with degree in the ingredient - target network

**No.**	**Compounds**	**Molecular formula**	**M (Da)**	**Degree**
**1**	D-Tetrandrine	$C_{38}H_{42}N_2O_6$	622.75	77
**2**	Paeonilactone C	$C_{17}H_{18}O_6$	318.32	72
**3**	Quercetin	$C_{15}H_{10}O_7$	301.04	68
**4**	(Z)-3-(2-Hydroxybutylidene)isobenzofuran-1(3H)-one	$C_{12}H_{12}O_3$	204.22	67
**5**	Levistilide A	$C_{24}H_{28}O_4$	380.48	63
**6**	senkyunolide O	$C_{24}H_{28}O_4$	380.48	63
**7**	inokosterone	$C_{27}H_{44}O_7$	481.32	62
**8**	Maslinic acid	$C_{30}H_{48}O_4$	471.35	62
**9**	Corosolic acid	$C_{30}H_{48}O_4$	471.35	59
**10**	Ginsenoside Rk3	$C_{36}H_{60}O_8$	621.43	58
**11**	Kaempferol	$C_{15}H_{10}O_6$	287.05	58
**12**	CALYCOSIN	$C_{16}H_{12}O_5$	285.08	58
**13**	coniferyl ferulate	$C_{20}H_{20}O_6$	356.37	57
**14**	Luteolin	$C_{15}H_{10}O_6$	287.05	57
**15**	Albiflorin	$C_{23}H_{28}O_{11}$	480.46	55
**16**	Ferulic acid	$C_{10}H_{10}O_4$	194.18	54

Continued Table. S3

**17**	**Paeoniflorin**	**$C_{23}H_{28}O_{11}$**	**480.47**	**51**
**18**	Ligustilide/Z-Ligustilide	$C_{12}H_{14}O_2$	190.24	48

Figure S1 The positive and negative ion flow diagram marked by standard samples.

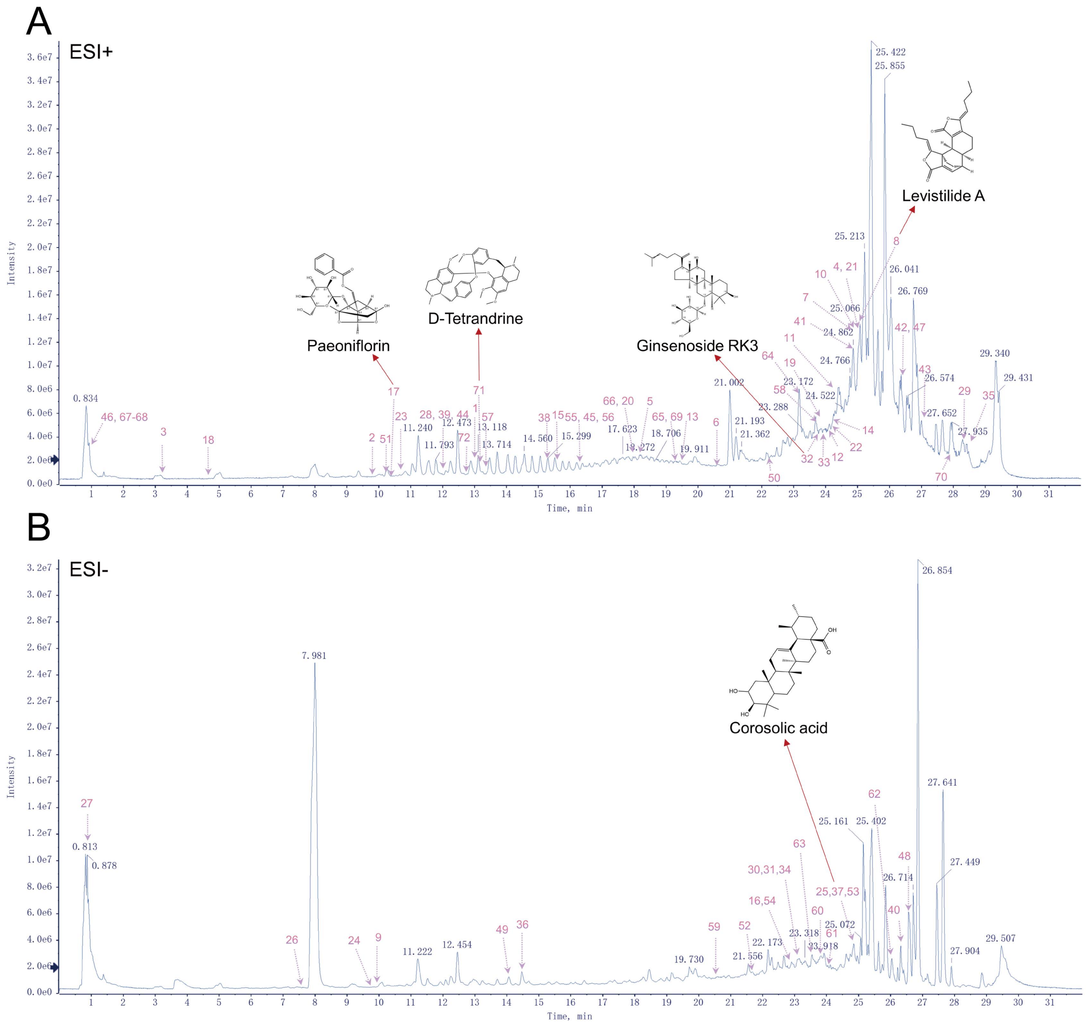

Figure S2

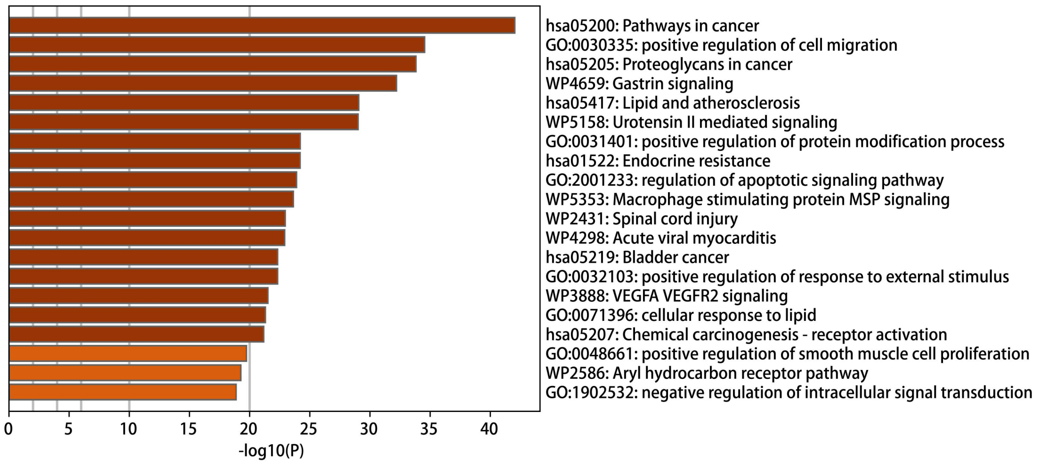

Figure S3

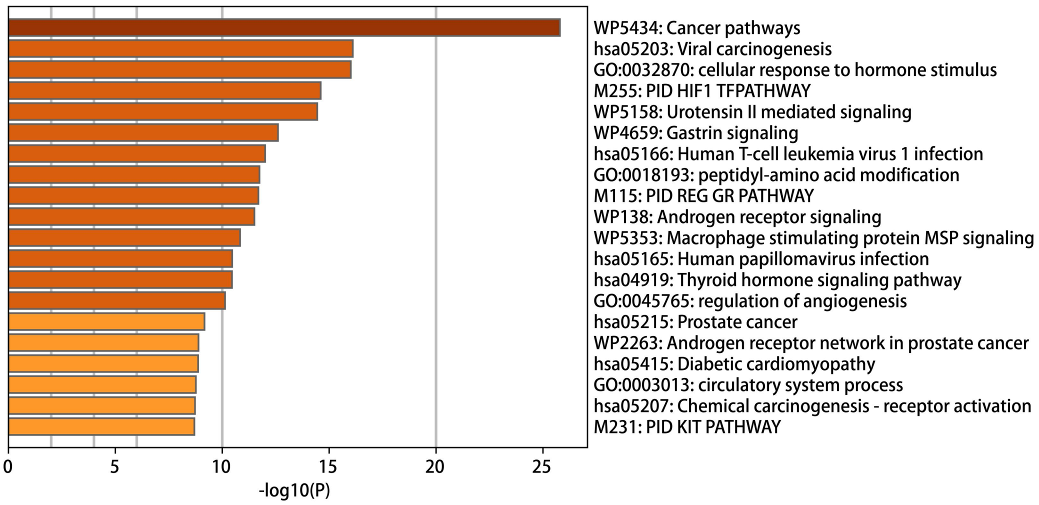

Supplement: Supplementary file 1 — Supplementary Material 1 [file 13020_2025_1220_MOESM1_ESM.pdf]
